# Novel Bioequivalent Sitagliptin and Metformin Bilayer Tablet with Improved Chemical Stability

**DOI:** 10.3390/pharmaceutics18091066

**Published:** 2026-08-26

**Authors:** In Gyu Yang, Jun-Young Han, Min Young Jeong, Dong-Wan Seo, Myung Joo Kang, Sun Ho Kim

**Affiliations:** 1 College of Pharmacy, Dankook University, 119 Dandae-ro, Dongnam-gu, Cheonan 31116, Chungnam, Republic of Korea; inq72220320@dankook.ac.kr (I.G.Y.); gkswnsdud@navipt.com (J.-Y.H.); jmy951207@dankook.ac.kr (M.Y.J.); dwseomb@dankook.ac.kr (D.-W.S.); 2 College of Pharmacy, Wonkwang University, 460 Iksan-daero, Iksan 54538, Jeonbuk, Republic of Korea

**Keywords:** bilayer tablet, metformin, sitagliptin, stability, dissolution, pharmacokinetics

## Abstract

**Objectives**: Fixed-dose combination tablets containing sitagliptin hydrochloride (SG) and metformin hydrochloride (MF) are a mainstay in the clinical management of type 2 diabetes. However, SG is highly susceptible to chemical degradation during storage, particularly in the presence of MF. Herein, a bilayer tablet in which SG and MF are physically separated into distinct layers was designed to enhance the chemical stability of SG while ensuring pharmacokinetic equivalence to the marketed reference product Janumet^®^. **Methods**: The compositions of individual SG and MF compartments were selected based on evaluations of their physical properties and dissolution profiles. Critical process parameters, including the pre- and main compression forces and coating levels of bilayer tablets, were fine-tuned to achieve dissolution characteristics comparable to those of the reference product. **Results**: Under accelerated storage conditions (40 °C, 75% relative humidity), the optimized bilayer tablet exhibited superior stability, with total SG-related impurity levels of 0.18% compared with 0.86% in the reference product after six months. Furthermore, in a randomized bioequivalence study in healthy volunteers (*n* = 30), the SG/MF bilayer tablet was pharmacokinetically equivalent to the reference product, with all parameters falling within the Food and Drug Administration-mandated regulatory criteria. **Conclusions**: In conclusion, this SG/MF bilayer tablet has better storage stability than the reference product and may be an alternative to conventional SG/MF combination tablets.

## 1. Introduction

Fixed-dose combination (FDC) therapy has emerged as a cornerstone in the clinical management of chronic diseases, particularly type 2 diabetes mellitus (T2DM), for which complex multidrug regimens are frequently necessitated. By consolidating multiple active pharmaceutical ingredients (APIs) into a single-dosage form, FDCs significantly mitigate “pill burden,” thereby enhancing patient treatment adherence and improving overall therapeutic outcomes [1]. Among the various synergistic combinations used for T2DM, the co-administration of a biguanide derivative and a dipeptidyl peptidase-4 (DPP-4) inhibitor is widely recognized for its superior glycaemic control and favourable safety profile [2].

Metformin hydrochloride (MF), a representative biguanide, remains the global gold standard first-line treatment for T2DM [3]. It exerts its antihyperglycemic effect by suppressing hepatic gluconeogenesis and increasing peripheral insulin sensitivity, while reducing the risk of hypoglycaemia and maintaining a weight-neutral profile [4]. MF has a high daily dose requirement (up to 2000 mg) and is primarily absorbed in the upper gastrointestinal tract, with a moderate oral bioavailability of 50–60% [5]. Sitagliptin hydrochloride (SG), a potent DPP-4 inhibitor, is frequently added to this regimen to improve glucose homeostasis by preventing the enzymatic degradation of incretin hormones, such as glucagon-like peptide-1 (GLP-1). This mechanism stimulates insulin secretion and suppresses glucagon release in a glucose-dependent manner [6]. Currently, several FDC products incorporating both of these agents into a monolithic matrix tablet are commercially available and widely utilized in clinical practice because SG/MF FDC tablets provide therapeutic convenience and have demonstrated bioequivalence to the concomitant administration of the individual components [7,8]. However, the formulation of these APIs into a conventional monolithic (single-layer) matrix presents significant pharmaceutical challenges, primarily regarding chemical stability while maintaining bioequivalent performance. SG is a chemically labile molecule; it is particularly prone to degradation under thermal and moisture stress [6]. In a shared matrix system including MF, the stability of SG is further compromised. This is due to the ability of MF, as a strongly basic biguanide, to elevate the local micro-environmental pH, which catalyses the deprotonation of the primary amine group of SG. This nucleophilic activation triggers deleterious drug–drug interactions and promotes the formation of various degradation products, such as *N*-acyl derivatives and cyclized adducts, during manufacturing and long-term storage [9,10,11]. In addition, SG stability is compromised by incompatibility with the lubricant sodium stearyl fumarate (SSF), which is commonly employed in MF/SG FDC tablets. Its primary amine undergoes a nucleophilic Michael addition with the fumarate moiety of SSF to form *N*-succinyl sitagliptin.

Multilayer tablets, including bilayer tablets (BLTs), represent an advanced solid oral dosage form that consolidates two distinct formulations into a single cohesive unit through a sequential compaction process. Beyond the primary benefit of reducing pill burden, the BLT architecture serves as a sophisticated engineering solution for managing complex drug–drug and drug–excipient interactions [12]. Furthermore, BLT systems enable the independent modulation of drug release profiles by targeting the individual layers [13]. Recent studies have demonstrated that the performance of BLTs is strongly governed by layer-specific formulation design, structural configuration, and process optimization, including Quality by Design (QbD)-based formulation strategies and hybrid manufacturing approaches [14,15]. Despite these advantages, BLTs are susceptible to mechanical failures, such as lamination or interfacial separation, which are often driven by differences in the elastic recovery and plastic deformation characteristics of the materials constituting the two layers during decompression [16]. To ensure structural integrity, it is paramount to adjust the pre-compression force (PRE-F) and main compression force (MAIN-F) accordingly. Precise modulation of PRE-F is required to facilitate sufficient granule fragmentation and provide a receptive surface for the second layer, while the MAIN-F predominantly affects the hardness, porosity, and release profile of multilayer tablets [17]. Beyond these mechanical constraints, establishing dual-drug compatibility to minimize physical or chemical interactions through a bilayer architecture, while simultaneously achieving pharmacokinetic bioequivalence of both active ingredients compared to their monolithic counterparts, remains highly challenging. Due to the substantial dose asymmetry between SG and MF, coupled with the restricted absorption of MF in the upper small intestine (the ‘absorption window’), achieving in vivo bioequivalence requires sophisticated formulation study [10]. While a previous study has documented a sitagliptin phosphate and MF bilayer formulation [11], the crucial influence of compaction force on mechanical strength, drug release behavior, and the chemical stability of BLT was not investigated. Consequently, critical insights into these formulation–process–performance interrelationships remain to be elucidated.

This study aimed to develop an SG/MF BLT with enhanced chemical stability and a bioequivalent pharmacokinetic (PK) profile compared with that of a monolithic matrix tablet (MRP; Janumet^®^ 50/1000 mg, Merck, Rahway, NJ, USA). To achieve this, the SG and MF compartments were separately prepared via direct compression and wet granulation, respectively, aligning their dissolution profiles with those of the MRP. These two compartments were subsequently consolidated into a BLT system, where the PRE-F, MAIN-F, and film-coating levels were fine-tuned to ensure adequate mechanical properties and achieve target release profiles. The chemical stability of the optimized BLT was evaluated under accelerated conditions (40 °C/75% RH) for 6 months. Furthermore, a randomized, crossover PK design and bioequivalence between BLT and MRP were evaluated in healthy adult subjects, with validated LC-MS/MS analysis.

## 2. Materials and Methods

### 2.1. Materials

SG hydrochloride and MF hydrochloride were obtained from Korea Biochem Pharm. Co., Ltd. (Sejong, Republic of Korea) and ABHILASHA PHARMA (Ankleshwar, India), respectively. The D_90_ (the particle diameter at which 90% of the cumulative volume is smaller than this value) values of SG and MF powder were 130.8 and 38.2 μm, respectively, as measured using a laser diffraction particle size analyzer (Mastersizer 3000; Malvern Panalytical, Malvern, UK). Microcrystalline cellulose (MCC; HEWETEN^®^ 102 and VIVAPUR^®^ 200) and SSF (PRUV^®^) were obtained from JRS Pharma (Rosenberg, Germany). Dicalcium phosphate dihydrate (DCPD; Di-Tab^®^) was obtained from Innophos (Cranbury, NJ, USA). Povidone K30 (Kollidon^®^ 30) was obtained from BASF (Ludwigshafen, Germany), and hydroxypropyl cellulose (HPC-L) was obtained from Nippon Soda (Tokyo, Japan). Croscarmellose sodium (Primellose^®^) was obtained from DFE Pharma (Goch, Germany), colloidal silicon dioxide (Aerosil^®^ 200) was obtained from Evonik (Essen, Germany), and magnesium stearate was obtained from Faci (Carasco, Italy). The film-coating material Tabshield Brown 21B907 was composed of polyvinyl alcohol (40.0%), titanium dioxide (17.6%), polyethylene glycol 3350 (20.2%), talc (14.8%), red iron oxide (6.0%), and yellow iron oxide (1.4%) and was obtained from Copitek (Suwon, Republic of Korea). The MRP, Janumet^®^ 50/1000 mg tablets (Merck, Rahway, NJ, USA), was purchased from Shindeok Pharmaceutical Co., Ltd. (Seoul, Republic of Korea; lot No. T035331 and U009951) and used for comparative dissolution and bioequivalence analyses. All solvents were of high-performance liquid chromatography grade.

### 2.2. Preparation of SG–MF-Loaded BLTs

The SG blend and MF granules were prepared by direct compression and wet granulation, respectively, as outlined in Table 1 and Table 2. For the SG blend, the SG powder was sieved through a 425-µm screen. MCC, DCPD, and binder (povidone K30) were accurately weighed and premixed in a polyethylene bag for 5 min. Magnesium stearate sieved through a 425-µm screen was added to the bag, and the mixture was lubricated for 10 min. To prepare the MF granules, the MF powder was first milled using an oscillator (RC60, Seoul High-Tech, Ansan, Republic of Korea) and passed through a 850-µm screen (850 µm). The MF powder and binder (HPC-L) were accurately weighed, added to the chamber of a high-speed mixer (SM-5C, Sejong, Incheon, Republic of Korea), and mixed for 3 min at an agitator speed of 130 rpm and a chopper speed of 3000 rpm. Subsequently, purified water (90 mg/tablet) was added to the mixture for over 30 s under mixing, and granulation was continued for 8 min. The wet granules were dried in a tray dryer (WOF-155, DAIHAN Scientific, Wonju, Republic of Korea) at 60 °C until the loss on drying (LOD) was ≤2.0% (*w*/*w*). Residual moisture was controlled using this LOD criterion, considering its reported influence on interparticulate bonding and the mechanical properties of high-dose MF tablets, including hardness and friability [18]. Then, the dried granules were sieved through a 850-µm sieve using an oscillator (RC60, Seoul High-Tech, Ansan, Republic of Korea). The sieved granules, diluent (MCC), disintegrant (croscarmellose sodium), and glidant (colloidal silicon dioxide) were sieved through a 425-µm sieve, accurately weighed, and mixed in a polyethylene bag for 10 min. To ensure blend uniformity, each component was accurately weighed, passed through the specified sieve, and mixed for a fixed time before lubrication. Subsequently, SSF was sieved through a 425-µm sieve, added to the mixture, and lubricated for 10 min.

The compositions and physicochemical characteristics of the individual SG and MF compartments used for BLT preparation are summarized in Table 1 and Table 2, respectively. The tablets were prepared in laboratory-scale batches (1000 tablets). The SG and MF compartments were compressed using a single-layer rotary tablet press (ZP-8, Shanghai Tianhe, Shanghai, China), whereas the BLTs were manufactured using a bilayer rotary tablet press (KT08SS-2L, Keumsung, Ansan, Republic of Korea). The single-layer rotary tablet press was equipped with Euro D-type convex punches (Changseong Precision, Incheon, Republic of Korea), with 8.5 mm round punches for the SG compartments and oval punches (long axis, 21.43 mm; short axis, 10.48 mm) for the MF compartments. The bilayer rotary press was equipped with two Euro D-type oval convex punches (long axis, 21.43 mm; short axis, 10.48 mm) (Changseong Precision, Incheon, Republic of Korea). The SG and MF compartments were prepared individually by directly compressing the SG blend or MF granules without layering, at a compression force of 15–20 kN (264–353 MPa for SG and 85–113 MPa for MF), which was selected to provide acceptable hardness and disintegration without tableting defects, and a turret speed of 10 rpm. Subsequently, BLTs were manufactured by loading and pre-compressing the MF granules, loading the SG blend, and then compressing the two compartments. The PRE-F and MAIN-F were set at 0.5–6.5 kN (2.8–36.9 MPa) and 20–40 kN (113–227 MPa), respectively, and the turret speed of the rotary tablet press was 10 rpm. The tablets were coated using Tabshield brown 21B907 dissolved in purified water at 16.0 *w*/*w*%. The coating was applied using a coating pan (PKC-30, Pharmatech Korea, Hwaseong, Republic of Korea) with a pan rotation speed of 18 rpm and a pump speed of 5–7 rpm; the inlet air temperature was set at 70 °C, and the product temperature was maintained at 48.0–50.0 °C. The LOD of the tablet cores before coating and the film-coated tablets after coating was controlled at ≤2.0% (*w*/*w*).

### 2.3. Analysis of Drug Content and Degradation Products

The drug content in the SG–MF-loaded BLTs was quantitatively analysed using a validated HPLC system (LC-40D, Shimadzu, Kyoto, Japan) [19]. Briefly, 10 tablets were placed in a mixture of 0.1% phosphoric acid and acetonitrile (950:50, *v*/*v*; 1000 mL) and sonicated for 10 min to extract the active ingredient. The resulting solution was diluted 10-fold prior to HPLC analysis, and the suspension was filtered through a 0.45 μm membrane filter. The HPLC system was equipped with a System Controller (CBM-40), an Auto Sampler (SIL-40C), a Solvent Delivery Module (LC-40D), a UV-VIS detector (SPD-40), a Degassing Unit (DGU-405), and a Column Oven (CTO-40C), and was operated using LabSolutions (Shimadzu Corporation, Kyoto, Japan). The mobile phase comprised ammonium phosphate buffer (17 g/L NH_4_H_2_PO_4_; pH adjusted to 3.0 with phosphoric acid) and acetonitrile (85:15 *v*/*v*), which was delivered at a flow rate of 1.5 mL/min to an SCX column (INNO, 4.6 × 150 mm, 5 μm; Young Jin Biochrom Co., Ltd., Seongnam, Republic of Korea) maintained at 30 °C. The injection volume was 5 μL, and detection was performed at 205 nm. The retention times for SG and MF were 4.2 min and 2.5 min, respectively. Reference standards of SG (Sigma-Aldrich, Steinheim, Germany) and MF (Sigma-Aldrich, St. Louis, MO, USA) were used for the analysis. The contents of SG and MF were determined by comparing the peak areas of the sample solutions with those of the corresponding reference standard solutions.

For the analysis of degradation products, 10 tablets were placed in a mixture of 0.1% phosphoric acid and acetonitrile (950:50, *v*/*v*; 500 mL) for SG or in the corresponding mobile phase (1000 mL) for MF. The resulting solution was diluted 10-fold prior to HPLC injection. The suspension was centrifuged, and the supernatant was filtered through a 0.45 μm membrane filter prior to HPLC injection. The same HPLC system described above was used for the analysis of degradation products (LC-40D, Shimadzu, Kyoto, Japan). The SG- and MF-related substance analyses were performed with reference to the corresponding USP–NF related-substance methods [20]. For SG-related substances, the mobile phase comprised phosphate buffer (1.36 g/L KH_2_PO_4_; adjusted to pH 2.0 with phosphoric acid) and acetonitrile (85:15, *v*/*v*), which was delivered at a flow rate of 1.0 mL/min to a cyano column (Discovery^®^, 4.6 × 150 mm, 5 μm; Supelco, Bellefonte, PA, USA) maintained at 30 °C. The injection volume was 20 µL, and detection was performed at 205 nm for 60 min. For MF-related substances, the mobile phase comprised ammonium phosphate buffer (17 g/L NH_4_H_2_PO_4_; adjusted to pH 3.0 with phosphoric acid), which was delivered at a flow rate of 1.5 mL/min to an SCX column (INNO, 4.6 × 250 mm, 5 μm; Young Jin Biochrom Co., Ltd., Seongnam, Republic of Korea) maintained at 40 °C. The injection volume was 5 μL, and detection was performed at 218 nm for 40 min. Related impurity peaks, including pharmacopeial related substances described in the USP–NF monographs, were monitored during stability testing [20]. Major SG-related degradation products were identified using the corresponding reference standards based on retention time. Impurity levels were expressed as relative peak-area percentages. Total SG-related and MF-related impurities were calculated as the sum of the corresponding related impurity peaks detected in each chromatogram.

### 2.4. Flowability and Compressibility of Powders

The bulk density (BD) was measured by carefully transferring 20 g of the powder into a 100-mL graduated cylinder and was calculated using Equation (1). The tapped density (TD) was evaluated by transferring 100 g of the sample into a 250-mL graduated cylinder. The initial volume (*V*_1_) was recorded, and the cylinder was tapped 1250 times using a tap density tester (JV 2000, COPLEY, Nottingham, UK). The final tapped volume (*V*_2_) was recorded, and TD was calculated using Equation (2). The Hausner ratio, an index of powder flow properties, was obtained from the ratio of TD to BD using Equation (3). The compressibility index, also known as Carr’s index, was calculated according to the equation described in the United States Pharmacopoeia [21] using Equation (4).

(1) $$BD=\frac{W}{V_{1}}$$

(2) $$TD=\frac{W}{V_{2}}$$

(3) $$Hausner\;ratio=\frac{TD}{BD}$$

(4) $$Carr’\;sindex\;(\%)=\frac{TD-BD}{TD}\times 100$$

where *W* is the powder weight used for each measurement, *V*_1_ is the initial bulk volume, and *V*_2_ is the final tapped volume.

### 2.5. Mechanical Properties of Tablets

The mechanical properties of the tablets, including thickness, hardness, and friability, were evaluated according to the USP guideline [22]. Tablet hardness (N) and thickness (mm) of the tablets were measured using a hardness tester (TBH 125; Erweka, Heusenstamm, Germany; *n* = 10 per formulation), and friability was determined using a friability tester (PT F20E; Pharma Test, Hainburg, Germany). Friability was measured once for each formulation using one set of 10 tablets, following the general procedure described in USP [23]. Accurately weighed tablets (*n* = 10) were placed in the friability tester and rotated at 25 rpm for 4 min. Loose dust was removed, the tablets were reweighed, and friability was calculated as the percentage weight loss (%) relative to the initial tablet weight.

### 2.6. Disintegration Time of Tablets

The disintegration time of the tablets was evaluated according to the USP method using a disintegration tester (KJ-DIT-200, Kukje Engineering, Paju, Republic of Korea) [24]. The disintegration apparatus comprised a basket-rack assembly, a 1000 mL beaker, a thermostatic arrangement for warming the medium, and a mechanical device to raise and lower the basket into and out of the immersion fluid at a constant frequency. Six tablets (one tablet per tube) were placed in 900 mL of water maintained at 37 ± 0.5 °C, and the disintegration time was determined visually.

### 2.7. In Vitro Dissolution Profile of Tablets

The in vitro dissolution profiles of the tablets were evaluated using the USP Apparatus II paddle method with a dissolution tester (DST-810, Labfine, Anyang, Republic of Korea) as an in vitro quality evaluation to compare the formulations with the MRP under consistent test conditions [25]. The pH 1.2 dissolution medium was prepared by dissolving 2.0 g of sodium chloride and 7.0 mL of hydrochloric acid in water and diluting to 1000 mL, of which 900 mL was maintained at 37 ± 2 °C for dissolution testing. Then, the tablets were added to the vessels and agitated at a paddle speed of 50 rpm. At predetermined time points, 5 mL aliquots were filtered through a syringe filter (RC, 0.45 µm). The filtrates were analysed using the HPLC assay method described in the Section 2.3. The similarity factor (*f*_2_) was calculated to compare the dissolution profiles of the selected formulations with that of the MRP. An *f*_2_ value of 50 or greater was considered to indicate similarity between two dissolution profiles.

### 2.8. Storage Stability of SG–MF-Loaded BLTs Under Accelerated Conditions

The physicochemical stability of the SG–MF-loaded BLTs was assessed under accelerated storage conditions (40 °C and 75% relative humidity) in accordance with the International Council for Harmonisation guidelines [26]. The film-coated BLTs were packaged as press-through packages (PTPs) using Triplex (Zymax TX, Bilcare, Pune, India) with a packaging machine (XENA-III, Raon Xena, Siheung, Republic of Korea) in a stability chamber (STH-305, DAIHAN Scientific, Wonju, Republic of Korea). For MRP, the commercial PTP tablets were used and stored together with the BLTs. Stability was evaluated at 0, 3, and 6 months to monitor changes in product quality. Degradation products were analysed using the HPLC impurity analysis method described in the Section 2.3.

### 2.9. PK and Bioequivalence Evaluations in Healthy Subjects

#### 2.9.1. Subjects and Drug Administration

The PK profile and bioequivalence of the SG–MF-loaded BLTs were evaluated in healthy adult volunteers and compared with those of the MRP. The study protocol was approved by the Institutional Review Board of H Plus Yangji Hospital on 14 April 2022 (IRB No. 2022-04-002-016), and formally authorized by the Ministry of Food and Drug Safety (MFDS), Republic of Korea, on 12 May 2022 (reception number: 20220091455). The study adhered to the Declaration of Helsinki and ICH Good Clinical Practice guidelines. The first subject was screened on 8 June 2022, and the last subject completed the final observation on 5 July 2022.

All participants voluntarily provided written informed consent after receiving comprehensive information regarding the study’s objective, procedures, and potential adverse events [27]. Eligible candidates were healthy adults aged ≥19 years with a body weight of ≥50.0 kg for males and ≥45.0 kg for females, and a body mass index between 18.0 and 30.0 kg/m^2^. Subjects had no clinically significant congenital or chronic medical conditions and were deemed eligible based on baseline physical examinations, electrocardiograms, and clinical laboratory tests (hematology, blood chemistry, serology, and urinalysis). Key exclusion criteria included: (1) pregnant or lactating females; (2) history of gastrointestinal disorders or surgical interventions that could alter drug absorption; (3) administration of drug-metabolizing enzyme inducers or inhibitors (e.g., barbiturates) within 1 month, or any medication that could interfere with the study outcome within 10 days prior to the first dose; (4) participation in another clinical trial within 6 months prior to study initiation; (5) a history or presence of clinically significant systemic diseases; (6) excessive alcohol consumption (>21 units/week for males; >14 units/week for females) or heavy smoking (>20 cigarettes/day) within 1 month prior to dosing; and (7) known hypersensitivity to the study drugs or any condition that could impair drug disposition or protocol compliance. Relevant regulatory information is publicly accessible on the official MFDS clinical trial registry (official clinical trial record, Available online: https://nedrug.mfds.go.kr/pbp/CCBBC01/nexacroPageOpen?approvalEnd=2026-08-24&approvalDtStart=2022-05-12&searchType=ST3&searchYn=true&localList2=000&localList=000&approvalStart=2023-08-24&approvalDtEnd=2022-05-12&page=1&&clinicExamSeq=202200418&clinicExamNo=100827&receiptNo=20220091455&approvalDt=2022-05-12 (accessed on 15 August 2026)).

PK and bioequivalence studies were conducted using an open-label, randomized, single-dose, two-period crossover design. Thirty subjects were randomly assigned to two groups: in each study period, the MRP or test product was administered at approximately 8:00 AM with 150 mL of water following an overnight fast of at least 10 h. All subjects consumed the same dinner on the day before dosing. Water intake was not permitted for 1 h before and after drug administration. At 0, 0.25, 0.5, 0.75, 1, 1.5, 2, 3, 4, 6, 8, 12, 24, and 34 h, 8 mL of blood samples were collected into K_2_-EDTA-treated tubes. The samples were centrifuged at 2200× *g* for 10 min at 2–8 °C to separate plasma, and the obtained plasma samples were stored below −80 °C until analysis. After a 7-day washout period, the alternate formulation was administered, and the same protocol was repeated.

#### 2.9.2. LC-MS/MS Analysis of SG and MF Concentrations in Human Plasma

Plasma concentrations of SG and MF were quantified using an Agilent 1260 series^®^ chromatography system (UPLC, Agilent, Santa Clara, CA, USA) coupled with an API 4000^®^ triple quadrupole mass spectrometer (AB SCIEX, Framingham, MA, USA), operating in multiple reaction monitoring (MRM) mode. Chromatographic separation was performed using a cyano column (Zorbax SB-CN, 150 × 4.6 mm, 5.0 μm; Agilent, Santa Clara, CA, USA) with a mobile phase consisting of methanol and 5 mM ammonium formate (80:20, *v*/*v*), delivered at a flow rate of 0.8 mL/min. The column oven was maintained at 30 °C, and the autosampler was maintained at 10 °C. The injection volume was 2 µL. Mass spectrometric detection was conducted in positive electrospray ionization mode. The MRM transitions were *m*/*z* 408.3 → 235.1 for SG and *m*/*z* 412.3 → 239.2 for the internal standard (ST-d4), and *m*/*z* 130.1 → 60.2 for MF and *m*/*z* 136.1 → 60.2 for the internal standard (MF-d6).

#### 2.9.3. PK Parameters and Statistical Bioequivalence Analysis

The PK parameters were estimated using a noncompartmental analysis model constructed with Phoenix™ WinNonlin^®^ (version 7.0; Certara USA, Inc., Princeton, NJ, USA). For each subject, the maximum plasma concentration (C_max_) and the time to reach C_max_ (T_max_) were determined from the individual plasma concentration–time data, and the resulting values were summarized as the arithmetic mean ± standard deviation (SD). The area under the plasma concentration–time curve from time zero to the last measurable time point (AUC_0–t_) was calculated using the linear trapezoidal approach to represent the systemic exposure to the drug. The total drug exposure after the last sampling point was determined by calculating the area under the curve from time zero to infinity (AUC_0–∞_) as the sum of AUC_0–t_ and the extrapolated portion C_t_/K_e_, where C_t_ is the plasma concentration at the last measurable time point, and K_e_ is the elimination rate constant. K_e_ was obtained from the terminal log-linear portion of the concentration–time curve, and t_1/2_ was determined using t_1/2_ = 0.693/K_e_, indicating the time required for the plasma concentration to decrease by half during the elimination phase.

Bioequivalence between the BLT and the MRP was evaluated by comparing log-transformed AUC_0–t_ and C_max_ values. The geometric mean ratios of BLT to the MRP were obtained, and 90% confidence intervals (CIs) were determined using the K-BE Test 2007 (version 1.1.0; Ministry of Food and Drug Safety [MFDS], Cheongju, Republic of Korea) [28]. Bioequivalence was achieved when the CIs for the log-transformed PK parameters were within the acceptance range of 80–125%, as defined by the FDA [29].

### 2.10. Statistical Analysis

Statistical analyses were performed using Minitab Statistical Software (version 22; Minitab LLC., State College, PA, USA). For dissolution profile comparison, the similarity factor (*f*_2_) was calculated to assess the similarity or distance between the selected formulations and the MRP [30].

## 3. Results

### 3.1. Formulation of the SG Compartment

In this study, SG/MF BLTs were rationally designed by first optimizing the individual SG and MF layers based on their respective physicochemical properties and dissolution profiles. Subsequently, PRE-F, MAIN-F, and coating levels were systematically adjusted to achieve drug release profiles comparable to those of the commercially available monolithic product. To develop the SG/MF BLTs, the individual compartments for SG and MF were first formulated based on their physicochemical properties and dissolution profiles. Subsequently, PRE-F, MAIN-F, and coating levels were adjusted to align the drug release profile with that of the commercially available monolithic product.

The SG compartment was prepared via direct compression based on prior knowledge of excipient functionality and preliminary compatibility and manufacturability screening. MCC and DCPD were selected as directly compressible fillers based on their compactability and dilution capacity, whereas povidone K30 and magnesium stearate were incorporated as a binder and lubricant, respectively, to ensure adequate tablet strength, powder flow, and tableting performance. The concentrations of these excipients were adjusted (SG1–SG6) to achieve excellent flowability and compactability while providing a dissolution profile comparable to that of the MRP. The BD and TD of the SG blend ranged from 0.526 to 0.714 g/mL and 0.645 to 0.870 g/mL, respectively. The Carr’s Index values, ranging from 17.9% to 22.9%, indicated ‘fair-to-passable’ flowability suitable for high-speed tableting [21]. The resulting tablets (SG1–SG6) maintained a hardness of 100.0–160.8 N, an acceptable drug content (98.5–101.6%), and low friability (≤0.05%), demonstrating robust physical integrity (Table 1).

**Figure 1:**
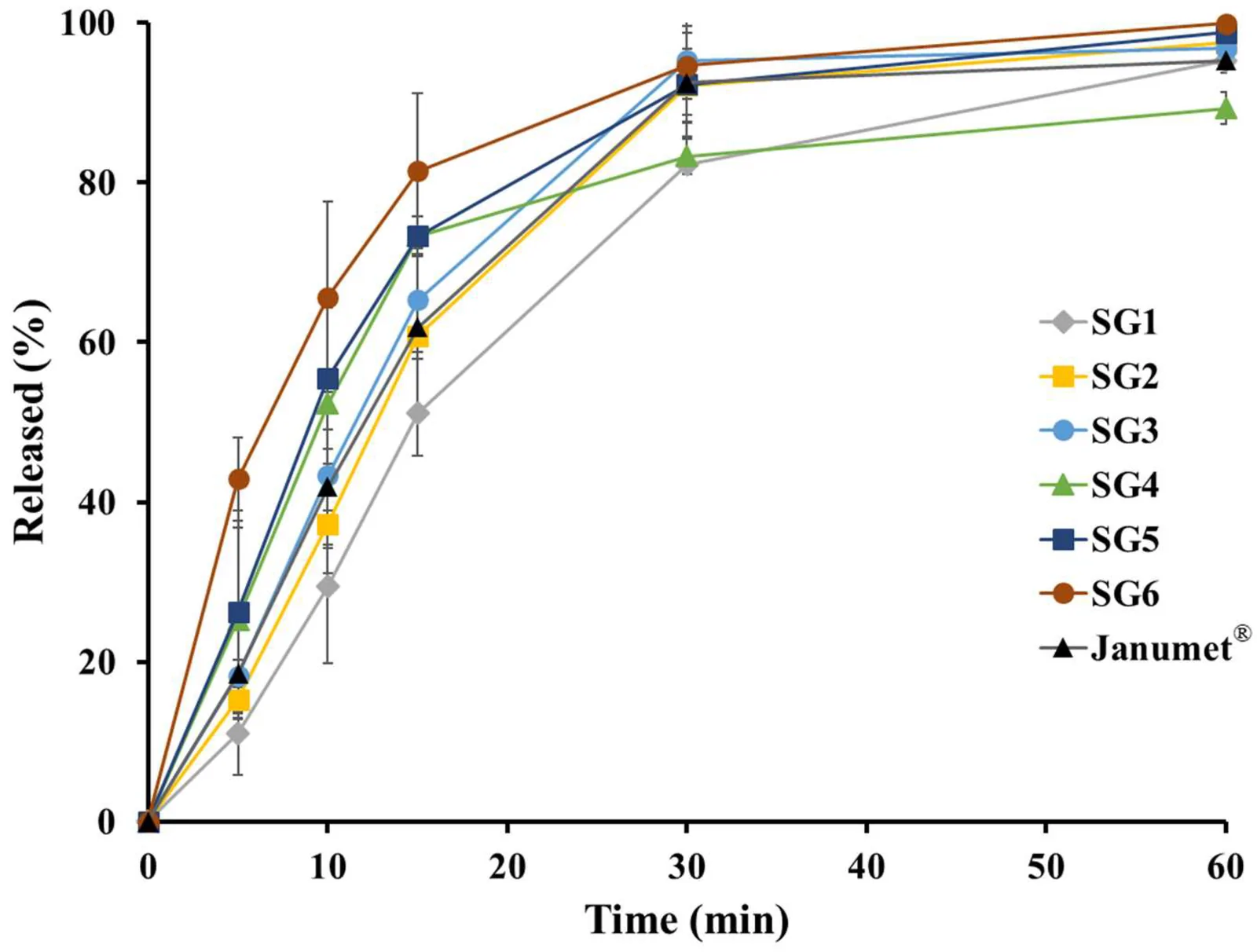
In vitro dissolution profiles of SG from single SG compartments and the MRP (Janumet^®^, 50/1000 mg) in the simulated gastric fluid (pH 1.2). Note: Data represent the means ± SD (*n* = 4).

In vitro dissolution tests were conducted at simulated gastric fluid (pH 1.2), because both SG and MF exhibit high solubility across the physiological pH range [31,32]. The MRP exhibited rapid release (Figure 1), with approximately 60% released within 15 min and over 90% within 30 min. The initial formulation, SG1, released 51.1% and 82.3% of the drug at 15 and 30 min, respectively, showing a slower profile compared to the MRP. To enhance the dissolution rate, the amount of MCC (VIVAPUR^®^ 200)—a binder/filler with high compactability [33]—was gradually reduced from 113.3 mg (SG1) to 20.0 mg (SG4). MCC plastically deforms during compression and increases interparticle bonding, thereby contributing to the strength and cohesiveness of compacts. Therefore, the reduction in MCC content may have decreased MCC-driven compact formation, as reflected by the decrease in tablet hardness from 160.8 N (SG1) to 100.0 N (SG4). This lower compact strength likely facilitated liquid penetration and tablet breakup. As the MCC content decreased, the disintegration time significantly shortened from 405 s (SG1) to 87 s (SG4). Correspondingly, the dissolution rates of SG1–SG3 increased. However, SG4 exhibited an unexpected retardation in dissolution after 30 min (83.3% at 30 min and 89.3% at 60 min). This phenomenon may be associated with a coning-like effect, as a mound of blend was visually observed at the bottom of the dissolution vessel. The relatively high density and non-swellable nature of DCPD could have contributed to this behavior [34], although further hydrodynamic evaluation would be required to confirm the exact mechanism. To minimize this potential effect, the DCPD fraction was reduced, and the MCC content was increased to 50 mg (matching the SG3 level) to maintain the target tablet weight. Furthermore, to enhance the dissolution kinetics, the concentration of povidone K30 was reduced from 6 mg (SG3) to 4 mg (SG5) and 2 mg (SG6). While povidone K30 serves as a robust binder, higher concentrations of this water-soluble polymer may have contributed to slower drug release by increasing local viscosity around the wetted blend, although the exact mechanism was not directly confirmed in this study [35]. SG6 exhibited markedly accelerated disintegration (83 s), yielding dissolution rates of 81.4% at 15 min and 94.6% at 30 min. Although the calculated similarity factor between SG6 and the MRP was 37.44, reflecting the faster initial release of SG6 during the first 15 min, the dissolution profiles became comparable by 30 min. SG has been reported to be well absorbed after oral administration, with an absolute bioavailability of approximately 87% and a T_max_ of 1–4 h, and its pharmacokinetics are largely unaffected by food intake [31,36,37,38]. Given these favourable biopharmaceutical and pharmacokinetic properties, the transient early divergence in SG dissolution was not expected to result in clinically meaningful differences in absorption. Therefore, SG6 was selected as the optimal SG compartment for the preparation of BLTs.

### 3.2. Formulation of the MF Compartment

The MF formulations were prepared by wet granulation based on prior knowledge and preliminary manufacturability screening, considering the high drug loading and poor tableting tendency of MF. HPC-L and povidone K30 were evaluated as binders, while MCC, croscarmellose sodium, colloidal silicon dioxide, and SSF were incorporated as a diluent, disintegrant, glidant, and lubricant, respectively, to ensure adequate granule flow, tablet strength, disintegration, and tableting performance. The MF compartment was engineered to provide a dissolution profile comparable with that of the MRP. To overcome the inherent propensity of MF for agglomeration [39], the drug was milled (850-µm) to ensure a uniform particle size distribution. Given its high drug loading (~85%), wet granulation was employed to enhance interparticle cohesion and mitigate capping, a common failure mode in high-load formulations driven by excessive elastic recovery during decompression [40]. MF granules exhibited ‘fair-to-passable’ flowability (Carr’s index: 15.2–17.8%) (Table 2), which is suitable for tableting [21]. SSF was used as a lubricant at a fixed level of 23 mg (1.8–2.0 wt.% of the MF compartment). Initial formulations (MF1 and MF2) using low binder levels were prone to capping due to weak bonding. This was resolved in MF3 by utilizing 40 mg of HPC-L, which promoted plastic deformation and yielded robust tablets (hardness: 169.7 ± 7.8 N) without mechanical defects [41].

**Figure 2:**
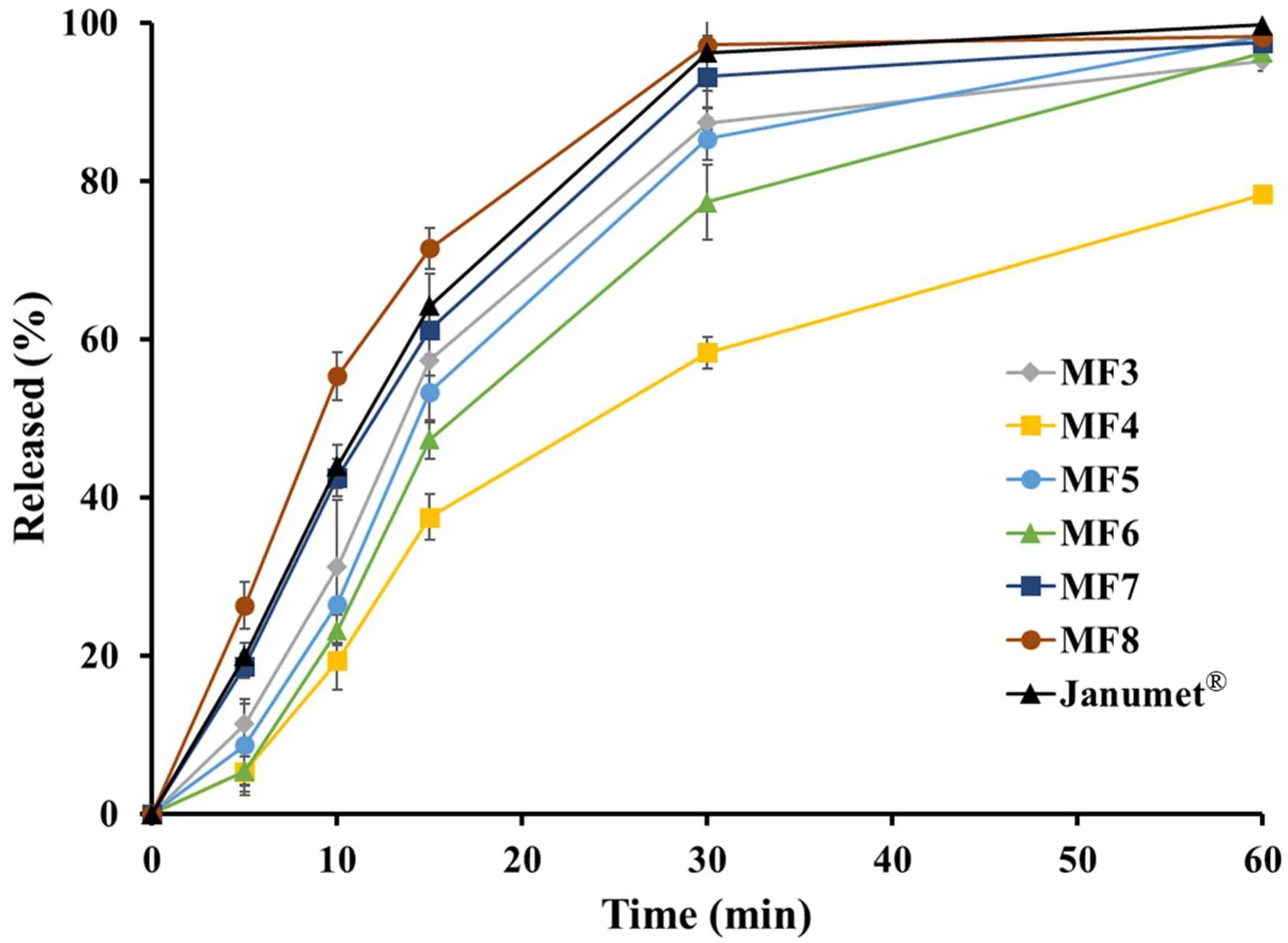
In vitro dissolution profiles of MF tablets and the MRP (Janumet^®^, 50/1000 mg) in simulated gastric fluid (pH 1.2). Note: Data represent the means ± SD (*n* = 4).

The in vitro dissolution profile of MF compartments was evaluated under simulated gastric fluid (pH 1.2) (Figure 2). While increasing the HPC-L content to 60 mg (MF4) further enhanced mechanical strength, it markedly retarded drug release (58.3% at 30 min) due to the formation of a viscous hydrated gel layer that hindered drug diffusion [42]. To optimise the dissolution rate, the HPC-L level was reduced back to 40 mg. Subsequent evaluations of insoluble filler levels (MF5 and MF6) revealed that excessive MCC may have reduced liquid penetration into the matrix, thereby impeding capillary liquid uptake and slowing disintegration [43]. Consequently, to make the profile comparable with that of the MRP, the superdisintegrant croscarmellose sodium was increased to 20 mg (MF7) and 35 mg (MF8). This adjustment significantly accelerated disintegration (7.8 min for MF8) and enhanced dissolution at 30 min to 97.2%, driven by the swelling and wicking action of croscarmellose sodium [44]. Although MF7 showed the closest dissolution profile to that of the MRP, MF8 still satisfied the similarity criterion, with a calculated *f*_2_ value of 55.12, and was selected for BLT fabrication to counterbalance the anticipated decrease in dissolution rate during high-pressure bilayer compaction and the subsequent film-coating process.

### 3.3. Formulation of BLTs

BLTs (target weight: 1370 mg) were fabricated by sequentially consolidating the MF8 granules as the first layer and the SG6 blend as the second layer. Tableting was performed using a pilot-scale rotary press with dimensions that matched those of the MRP. Process optimization focused on the PRE-F, MAIN-F, and coating levels to ensure mechanical robustness and dissolution profiles comparable to that of the MRP.

#### 3.3.1. Influence of PRE-F on Mechanical Properties and Dissolution

The influence of PRE-F [0.5–6.5 kN (2.8–36.9 MPa)] on the structural integrity and drug release of the BLTs was initially evaluated at a fixed MAIN-F of 30 kN (170 MPa) (Table 3 and Figure S1). At a low PRE-F [0.5 kN (2.8 MPa)], the BLTs exhibited a non-uniform interface between the MF and SG layers, accompanied by significant weight variation. This instability may be associated with insufficient consolidation of the first layer; specifically, the MF8 granules appeared to retain part of their granular morphology, resulting in an irregular and highly rough surface. Although moderate surface roughness may contribute to mechanical interlocking between adjacent layers, an excessively loose surface could be susceptible to disturbance by the second-layer powder feeder and the vacuum suction system, potentially leading to mass loss and interfacial defects [45]. Further evaluation of interfacial morphology and layer adhesion would be needed to clarify the role of interfacial interlocking in the structural integrity of the BLTs.

In contrast, a PRE-F of 3.5 kN (19.8 MPa) ensured superior mechanical properties without any observable tableting issues, while markedly reducing weight variation. This force facilitated adequate plastic deformation and fragmentation, providing a sufficiently flat yet receptive surface for the second layer [16]. This optimised consolidation promoted robust mechanical interlocking and interfacial adhesion between the MF and SG layers, ensuring the integrity necessary to maintain low friability (0.02%), even under the mechanical stress of the film-coating process. However, increasing the PRE-F to 6.5 kN (36.9 MPa) induced interfacial lamination. The over-densification of the first layer likely reduced the surface available for mechanical interlocking, thereby limiting the ability of the second-layer granules to anchor onto the first layer during the main compression phase [45].

**Figure 3:**
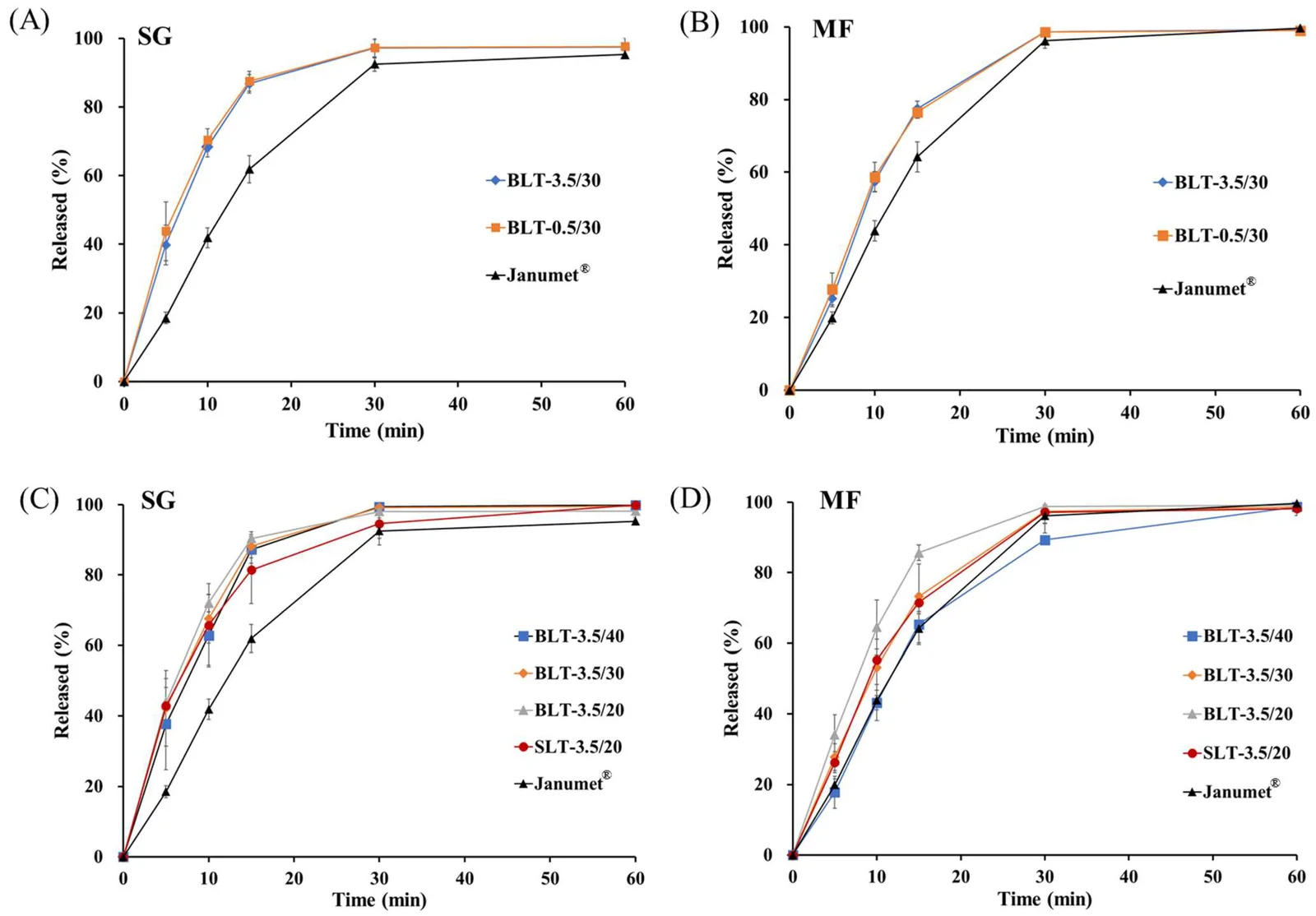
Impact of pre-compression force (PRE-F) and main compression force (MAIN-F) on the in vitro dissolution of (**A**,**C**) SG and (**B**,**D**) MF in BLTs compared with those of the MRP (Janumet^®^, 50/1000 mg) in simulated gastric fluid (pH 1.2). Note: Formulations are denoted as “formulation–PRE-F(kN)/MAIN-F(kN)”. In (**A**,**B**), PRE-F was altered [0.5 or 3.5 kN (2.8 or 19.8 MPa)] whilst MAIN-F was fixed at 30 kN (170 MPa). In (**C**,**D**), MAIN-F was altered [20–40 kN (113–227 MPa)] whilst PRE-F was fixed at 3.5 kN (19.8 MPa). SLTs were compressed at a PRE-F of 3.5 kN (19.8 MPa) and a MAIN-F of 20 kN (113 MPa). Data represent the means ± SD (*n* = 4).

Dissolution testing in simulated gastric fluid (pH 1.2, paddle method) revealed minimal differences between BLTs compressed at 0.5 kN (2.8 MPa) and 3.5 kN (19.8 MPa) (Figure 3A,B). At 15 min, SG release was between 86.8–87.5% and MF release ranged from 76.5–77.5%, both exceeding the initial release rate of the MRP. Thus, PRE-F primarily influenced mechanical integrity rather than dissolution kinetics.

#### 3.3.2. Effect of MAIN-F on the Mechanical Properties and Dissolution Profile of BLTs

The impact of MAIN-F [20–40 kN (113–227 MPa)] was investigated at a fixed PRE-F of 3.5 kN (19.8 MPa) (Table 4). As anticipated, increasing the MAIN-F led to a significant rise in tablet hardness (from 164.8 to 238.3 N) and corresponding prolongation of disintegration time (from 6.3 to 10.3 min). This may be attributed to greater tablet densification at higher compression forces, which enhances mechanical strength but limits liquid penetration and capillary uptake of the dissolution medium [46].

The dissolution behaviour exhibited drug-specific sensitivity to the MAIN-F. SG release remained relatively unaffected by the compression force (Figure 3C), likely due to the high aqueous solubility of SG and the relative thinness of the SG layer, which permits rapid media penetration regardless of the degree of densification [46]. Conversely, MF dissolution was significantly influenced by the MAIN-F (Figure 3D): increasing the force to 40 kN (227 MPa) significantly retarded MF release (89.3% at 30 min) compared to the 20–30 kN (113–170 MPa) range (>97%). This sensitivity may be associated with greater densification of the high-drug-load MF layer at higher compression forces, resulting in a more compact internal structure with fewer and less accessible interparticulate voids for penetration of the dissolution medium. In addition, the limited swelling behaviour of the formulation components may further delay liquid uptake and disintegration, thereby reducing MF release [47].

To further elucidate the effect of the bilayer structure, the dissolution profiles of the BLTs were compared with those of the individual SG6 and MF8 SLTs compressed at the same MAIN-F (Figure 3C,D). Overall, no significant differences were observed in the dissolution profiles of either SG or MF between the BLTs and SLTs throughout the dissolution period. For SG, the BLT–SLT comparisons showed *f*_2_ values of 66.10, 65.25, and 58.59 for BLT-3.5/40, BLT-3.5/30, and BLT-3.5/20, respectively. For MF, the corresponding *f*_2_ values were 51.97, 86.57, and 50.41, respectively. This may be attributed to the high solubility of both SG and MF and the rapid release characteristics of the formulations under pH 1.2 conditions, which likely minimised the influence of differences in surface area and tablet density between the BLTs and SLTs on the release rate. Ultimately, the BLT formulation prepared with a PRE-F of 3.5 kN (19.8 MPa) and a MAIN-F of 30 kN (170 MPa) was selected for the coating process, as it exhibited a dissolution profile comparable with that of the MRP for MF release, with an *f*_2_ value of 56.04.

#### 3.3.3. Effect of the Film-Coating Level on the Dissolution of SG–MF BLTs

The film coating acts as a protective physical barrier that modulates the initial water uptake kinetics of the tablet core. Increasing the coating level typically extends the induction period required for the dissolution medium to penetrate the core, thereby influencing both the disintegration time and the early-phase dissolution profile. In this study, a polyvinyl alcohol (PVA)-based immediate-release coating system was employed. As anticipated, the disintegration times for all film-coated BLTs were prolonged by approximately 2 min compared with those of the uncoated cores, regardless of the specific coating level within the tested range. This delay may be attributed to the time required for the film coating to hydrate before medium penetration into the tablet core [48].

**Figure 4:**
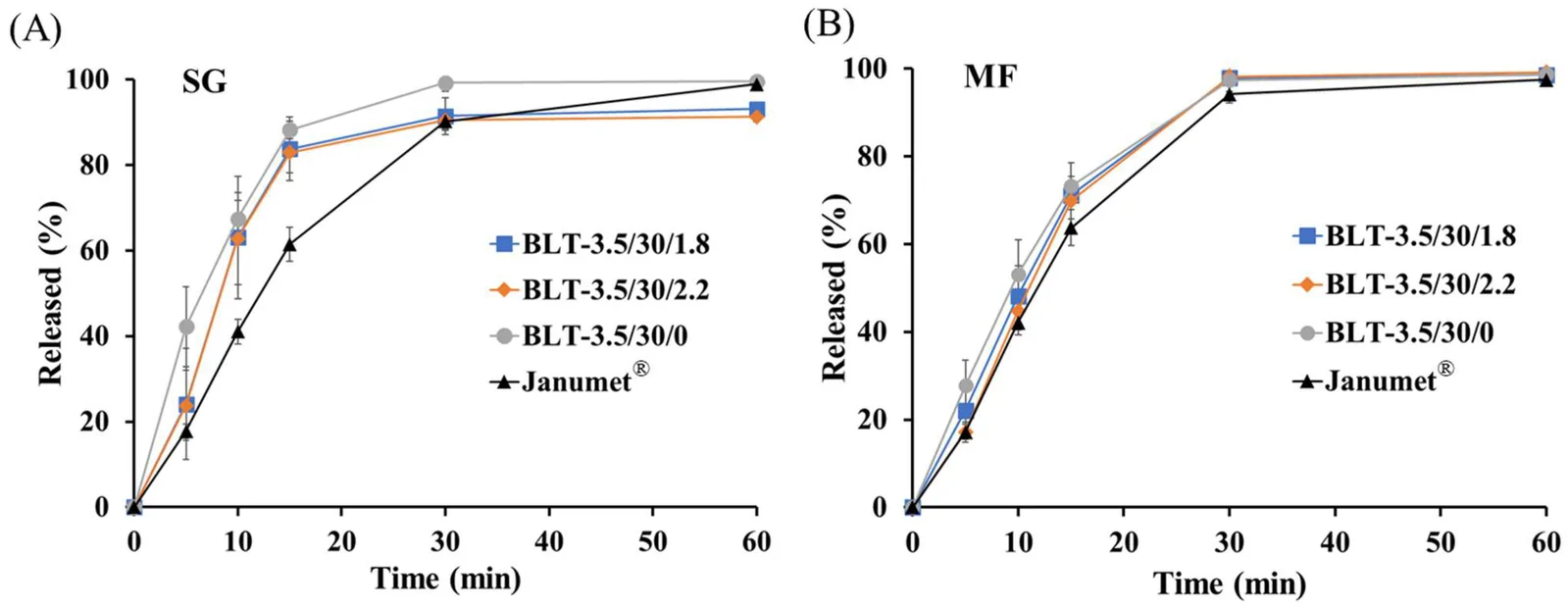
Effects of the coating level on the in vitro dissolution profiles of (**A**) SG and (**B**) MF from BLTs compared with those of the MRP (Janumet^®^, 50/1000 mg) in simulated gastric fluid (pH 1.2). Note: BLTs are denoted as “formulation–PRE-F(kN)/MAIN-F(kN)/Coating(%)”, where Coating(%) represents the percentage of coating weight gain. PRE-F and MAIN-F were fixed at 3.5 kN (19.8 MPa) and 30 kN (170 MPa), respectively. Evaluations included uncoated (0%) and film-coated tablets (1.8% and 2.2% weight gain). Data represent the mean ± SD (*n* = 4).

The dissolution profiles of the uncoated and coated BLTs were evaluated in simulated gastric fluid (Figure 4). While the coating markedly reduced SG release—indicating a delay in medium penetration and subsequent core disintegration—MF dissolution remained largely unaffected. The tablets with 1.8% and 2.2% coating levels both exhibited rapid initial SG release with nearly identical profiles (Figure 4A). Although the SG dissolution from the coated BLTs showed a transient divergence from that of the MRP during the initial dissolution phase, resulting in calculated *f*_2_ values of 37.08 and 37.69 for BLT-3.5/30/1.8 and BLT-3.5/30/2.2, respectively, the profiles became comparable by 30 min. Considering the favourable oral absorption characteristics of SG, including an absolute bioavailability of approximately 87%, a T_max_ of 1–4 h, and minimal food effect [31,36,37,38], this transient early divergence was not expected to result in clinically meaningful differences in absorption.

Moreover, the MF release profiles were highly comparable with that of the MRP regardless of coating level, with calculated *f*_2_ values of 60.88, 66.66, and 53.04 for BLT-3.5/30/1.8, BLT-3.5/30/2.2, and BLT-3.5/30/0, respectively (Figure 4B). This coating-dependent differential impact on SG and MF release can be attributed to the distinct release mechanisms of the two compartments. The dissolution of SG, located in the thinner layer, is more sensitive to the initial wetting kinetics and the rapid disintegration of the core. Conversely, the release of MF from the high-load compartment is predominantly governed by the internal matrix structure and disintegration behavior of the tablet core, making it less susceptible to the marginal lag time introduced by the film coating. Considering both dissolution similarity and coating appearance, the coating weight gain was ultimately set to 2.2% for the optimized BLT, because tablets coated to 1.8% weight gain exhibited visible mottling. Thus, the final optimised BLT was the formulation prepared at a PRE-F of 3.5 kN (19.8 MPa) and a MAIN-F of 30 kN (170 MPa), with film coating to a weight gain of 2.2%.

### 3.4. Stability of SG/MF BLT Under Accelerated Conditions

The development of FDC products often necessitates specialized formulation strategies to overcome chemical incompatibilities between active pharmaceutical ingredients. Physical separation designs, such as BLTs, have been successfully implemented for various incompatible pairs—including telmisartan–pravastatin and aspirin–clopidogrel—to minimize deleterious drug–drug contact and adverse microenvironmental interactions [49,50]. The rationale for adopting a BLT architecture for the SG–MF combination is primarily to account for the chemical vulnerability of SG. The stability of SG is fundamentally governed by the reactivity of its nucleophilic primary amine group, which is highly susceptible to degradation in alkaline or high-humidity environments. MF, as a strong basic biguanide derivative, can significantly elevate the local micro-pH within a shared tablet matrix. This alkaline micro-environment promotes the deprotonation of the SG primary amine, enhancing its nucleophilicity and triggering several distinct degradation pathways. Specifically, SG can undergo an intramolecular nucleophilic attack on its own carbonyl carbon to form a cyclized piperazinone derivative (Degradant B) or to form amide-linked degradants such as *N*-formyl sitagliptin (Degradant C). Furthermore, MF is hygroscopic and therefore tends to attract moisture, which can facilitate the hydrolysis of SG’s amide bond, resulting in the formation of Degradant A ((R)-3-amino-4-(2,4,5-trifluorophenyl)butanoic acid) [9,51,52].

**Figure 5:**
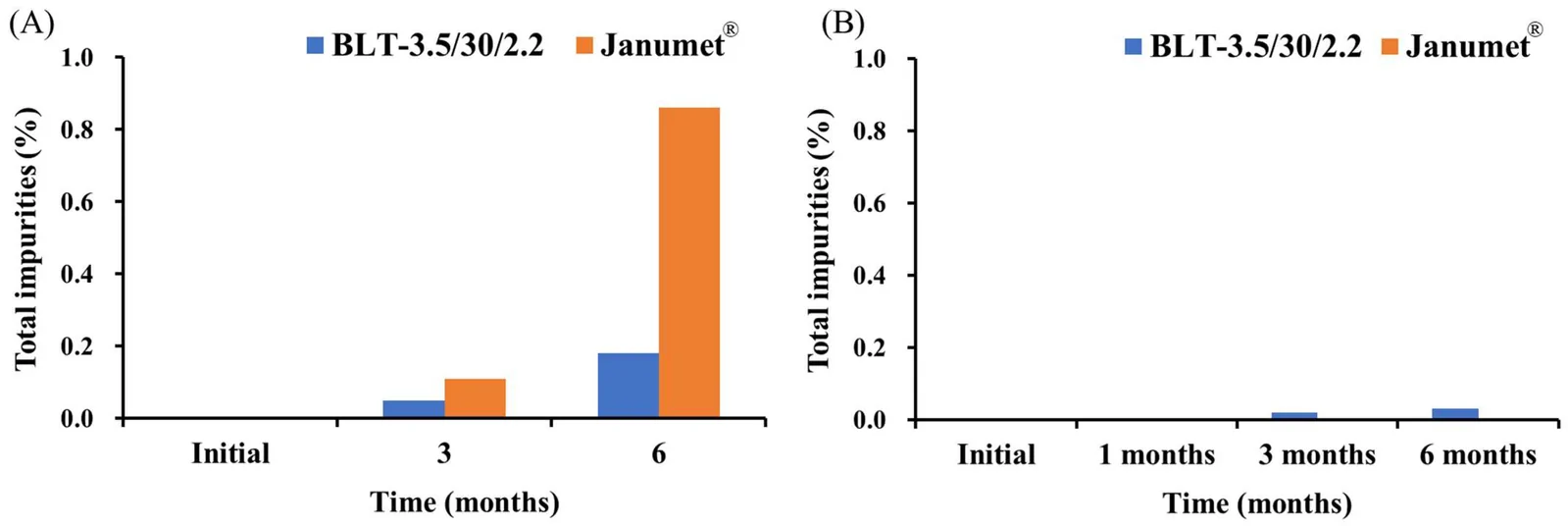
Comparison of total impurity levels originating from (**A**) SG and (**B**) MF between BLT and MRP (Janumet^®^, 50/1000 mg) under accelerated storage conditions (40 °C/75% RH). Notes: The total impurities were determined using a pooled sample of ten tablets (*n* = 1). Data represents the percentage of total impurities measured at 0-month, 3-month, and 6-month intervals.

The chemical stability of SG and MF in optimized BLT-3.5/30/2.2 was comparatively evaluated with that in the MRP under accelerated conditions (40 °C/75% RH) (Figure 5). While both the BLT and MRP exhibited 0.00% total SG-related impurities at the initial time point, a significant divergence emerged during storage. After six months, the total SG-related impurities in the MRP increased to 0.86%, whereas those in the BLT increased to only 0.18%. The USP monograph specifies a limit of NMT 0.6% for total degradation products in sitagliptin tablets [20]. The SG-related impurity profile was assessed using RRT-based peak assignment, and impurity peaks corresponding to sitagliptin acid (RRT 0.55), *N*-succinyl sitagliptin (RRT 1.2), sitagliptin phenylcrotonyl analog (RRT 4.1), and sitagliptin styrylacetyl analog (RRT 4.7) were monitored during accelerated storage. Among these RRT-based impurity peaks, those corresponding to sitagliptin acid and *N*-succinyl sitagliptin represented the major SG-related impurity peaks. The primary amine of sitagliptin undergoes a nucleophilic Michael addition with the fumarate moiety of SSF, forming *N*-succinyl sitagliptin [20,53]. In monolithic tablets, this degradation is considered to be accelerated by the hygroscopicity of MF. The increase in total SG-related impurities in the MRP was mainly associated with the increase in these dominant peaks, whereas their contribution was markedly lower in the optimized BLT. Therefore, the lower total SG-related impurity level in the BLT was mainly attributable to the reduced formation of the major RRT-based SG-related impurity peaks. This indicates that the spatial segregation of the two APIs markedly decreased SG degradation by preventing the elevation of the local micro-pH within the tablet matrix and mitigating the impact of MF’s inherent hygroscopicity.

In contrast, MF-related impurities remained negligible in both formulations, reaching a maximum of only 0.03% in the BLT and 0.00% in the MRP at six months, which is consistent with the inherent chemical stability of the MF molecule (Figure 5B) [54]. These results collectively demonstrate that the BLT-3.5/30/2.2 formulation provides a robust solution for maintaining the physicochemical stability of the SG–MF combination.

### 3.5. PK and Bioequivalence Evaluation in Healthy Subjects

**Figure 6:**
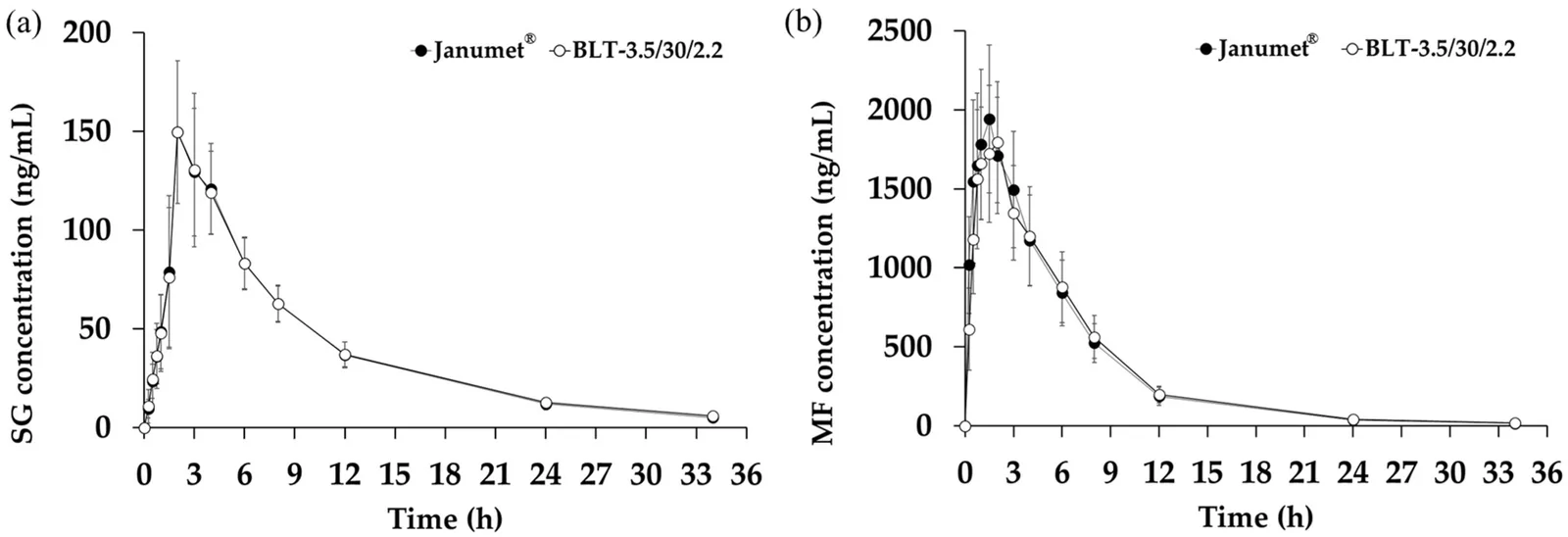
Plasma drug concentration–time profiles of SG and MF following oral administration of the MRP (Janumet^®^, 50/1000 mg, ●) and BLT (○) in healthy male subjects. (**a**) SG; (**b**) MF. Note: Data represent the mean ± SD (*n* = 30).

A randomized, single-dose, two-period crossover study was performed in healthy male subjects to compare the PK performance of the optimized BLT-3.5/30/2.2 with the MRP. The plasma concentration–time profiles and the derived PK parameters are presented in Figure 6 and Table 5, respectively. Following oral administration, the plasma SG concentration of the MRP increased rapidly, reaching a peak C_max_ of 150.6 ± 28.5 ng/mL at a T_max_ of 2.98 ± 0.81 h. Subsequently, SG plasma concentrations declined with a terminal half-life t_1/2_ of 7.74 ± 1.12 h. These findings are in excellent agreement with previously reported values for SG, which typically reflect rapid absorption (T_max_ of 1–4 h) and an elimination half-life of approximately 8–14 h [55,56]. BLT-3.5/30/2.2 exhibited a numerically comparable PK profile for SG, with a C_max_ of 151.1 ± 35.8 ng/mL, T_max_ of 3.10 ± 0.96 h. Total systemic exposure (AUC_0–34 h_) was also highly comparable, with values of 1283.1 ± 158.0 ng·h/mL for BLT and 1272.9 ± 180.2 ng·h/mL for the MRP. Notably, while the in vitro dissolution study showed a transient divergence at the 15-min mark (where SG release from the BLT was slightly more rapid than the MRP), this did not translate into a significant difference in vivo. Given that both formulations achieved nearly complete dissolution (>90%) within 30 min in the acidic environment (pH 1.2), SG was fully available in the upper gastrointestinal tract, leading to the observed bioequivalence [37].

Parallel PK trends were observed for MF (Figure 6b). The C_max_ for BLT-3.5/30/2.2 and the MRP were 1797.7 ± 373.1 ng/mL and 1959.6 ± 459.8 ng/mL, respectively, with corresponding T_max_ values of 1.73 ± 0.68 h and 1.40 ± 0.78 h (Table 5). The elimination phase remained consistent between the two products, with t_1/2_ values of 6.42 ± 1.67 h and 6.17 ± 1.37 h. The overall extent of absorption for MF was also comparable (AUC_0–34 h_ of 12,208.9 ± 2441.4 for BLT and 12,303.7 ± 2535.1 ng·h/mL for the MRP). As MF is a BCS Class III drug, excipient composition—particularly surfactants—can influence its intestinal absorption. Although the MRP contains sodium lauryl sulfate (SLS) while the BLT does not, the SLS level in the MRP is minimal and did not markedly affect the rate or extent of MF absorption. These results suggest that the high-load MF compartment in the bilayer structure successfully replicated the release characteristics of the monolithic MRP. The optimisation of the MF8 compartment, which utilized a balanced ratio of the superdisintegrant and binder, ensured that the densification from the bilayer tableting process did not impede the in vivo release or absorption of MF, which is primarily absorbed via saturable transporters in the proximal small intestine.

To establish bioequivalence, statistical analyses were conducted on log-transformed AUC_0–34 h_ and C_max_ values (Table 6). The test-to-reference (T/R) ratios of log-transformed AUC_0–34 h_ and C_max_ for SG were 1.01 and 0.99, respectively, and the corresponding 90% CI were 0.9864–1.0349 for AUC_0–34 h_ and 0.9458–1.0440 for C_max_, both of which were within the FDA-accepted bioequivalence range (0.80–1.25) [57]. Likewise, the T/R ratios of log-transformed AUC_0–34 h_ and C_max_ for MF were 0.99 and 0.92, respectively, and the corresponding 90% CI were 0.9440–1.0475 for AUC_0–34 h_ and 0.8636–0.9833 for C_max_, both of which were also within the bioequivalence range. Collectively, these results indicate that the SG–MF-loaded BLT-3.5/30/2.2 is bioequivalent to the MRP in terms of systemic exposure and the peak concentrations of SG and MF in healthy male subjects.

## 4. Conclusions

In the present study, a novel SG/MF BLT was successfully formulated, effectively overcoming the chemical stability challenges inherent in conventional monolithic matrix tablets. Following the optimisation of critical process parameters—including PRE-F, MAIN-F, and coating level—the BLT exhibited appropriate mechanical and physical properties and a dissolution profile comparable to that of the MRP, with no tableting defects. The established BLT achieved a 79% reduction in total SG-related impurities compared to the MRP after six months of storage under accelerated conditions (40 °C/75% RH), by achieving the spatial segregation of the two active ingredients. Furthermore, the SG/MF BLT demonstrated PK profiles for both SG and MF that were statistically equivalent to those of the reference product. This formulation strategy may also be applicable to other chemically incompatible fixed-dose combinations, in which a bilayer architecture can mitigate direct drug–drug contact and unfavorable microenvironmental interactions. In conclusion, the developed SG–MF BLT offers a promising approach for the stable and effective delivery of FDC, potentially improving patient safety and therapeutic outcomes in the management of type 2 diabetes.

## Figures and Tables

**Table 1:** Composition and physicochemical characteristics of the SG compartments.

	SG1	SG2	SG3	SG4	SG5	SG6
**Composition, mg/tablet (*w*/*w*%)**						
SG	56.7 (28.4)	56.7 (28.4)	56.7 (28.4)	56.7 (28.4)	56.7 (28.4)	56.7 (28.4)
MCC	113.3 (56.7)	83.3 (41.7)	50.0 (25.0)	20.0 (10.0)	50.0 (25.0)	50.0 (25.0)
DCPD	20.0 (10.0)	50.0 (25.0)	83.3 (41.7)	113.3 (56.7)	85.3 (42.7)	87.3 (43.7)
Povidone K30	6.0 (3.0)	6.0 (3.0)	6.0 (3.0)	6.0 (3.0)	4.0 (2.0)	2.0 (1.0)
Magnesium Stearate	4.0 (2.0)	4.0 (2.0)	4.0 (2.0)	4.0 (2.0)	4.0 (2.0)	4.0 (2.0)
Total (mg)	200.0	200.0	200.0	200.0	200.0	200.0
**B** **lend features**						
BD (mL, g/mL)	0.526	0.571	0.645	0.714	0.625	0.635
TD (mL, g/mL)	0.645	0.741	0.800	0.870	0.784	0.784
Carr’s index (%)	18.4	22.9	19.4	17.9	20.3	19.0
**Tablet features**						
Drug content (%) ^1^	100.3	101.1	101.3	98.5	101.6	98.5
Hardness (N) ^2^	160.8 ± 6.9	146.1 ± 5.9	130.4 ± 7.8	100.0 ± 5.9	119.6 ± 9.8	110.8 ± 10.8
Friability (%) ^3^	0.01	0.02	0.02	0.05	0.02	0.02
Disintegration (s) ^4^	405 ± 52	311 ± 62	197 ± 21	87 ± 7	145 ± 25	83 ± 13

Notes: Values in parentheses indicate *w*/*w*%. ^1^ The drug content was determined as the mean value of 10 tablets. ^2^ Data represent the means ± SD (*n* = 10). ^3^ Tablet friability was obtained from a single measurement of 10 tablets. ^4^ Data represent the means ± SD (*n* = 6).

**Table 2:** Composition and physicochemical characteristics of the MF compartments.

	MF1	MF2	MF3	MF4	MF5	MF6	MF7	MF8
**Composition, mg/tablet (*w*/*w*%)**								
MF	1000.0 (85.5)	1000.0 (85.5)	1000.0 (85.5)	1000.0 (85.5)	1000.0 (81.1)	1000.0 (76.9)	1000.0 (85.5)	1000.0 (85.5)
HPC-L	20.0 (1.7)	-	40.0 (3.4)	60.0 (5.1)	40.0 (3.2)	40.0 (3.1)	40.0 (3.4)	40.0 (3.4)
Povidone K30	-	40.0 (3.4)	-	-	-	-	-	-
MCC	107.0 (9.1)	87.0 (7.4)	87.0 (7.4)	67.0 (5.7)	152.0 (12.3)	217.0 (16.7)	72.0 (6.2)	57.0 (4.9)
Croscarmellose sodium	5.0 (0.4)	5.0 (0.4)	5.0 (0.4)	5.0 (0.4)	5.0 (0.4)	5.0 (0.4)	20.0 (1.7)	35.0 (3.0)
Colloidal silicon dioxide	15.0 (1.3)	15.0 (1.3)	15.0 (1.3)	15.0 (1.3)	15.0 (1.2)	15.0 (1.2)	15.0 (1.3)	15.0 (1.3)
SSF	23.0 (2.0)	23.0 (2.0)	23.0 (2.0)	23.0 (2.0)	23.0 (1.9)	23.0 (1.8)	23.0 (2.0)	23.0 (2.0)
Total	1170.0	1170.0	1170.0	1170.0	1235.0	1300.0	1170.0	1170.0
**Granule features**								
BD (mL, g/mL)	0.488	0.500	0.513	0.506	0.548	0.526	0.513	0.556
TD (mL, g/mL)	0.571	0.606	0.615	0.597	0.667	0.625	0.606	0.667
Carr’s index (%)	15.9	17.5	16.7	15.2	17.8	15.8	15.4	16.7
**Tablet features**								
Drug content (%) ^1^	100.0	98.8	99.7	101.2	100.3	100.1	98.7	99.5
Hardness (N) ^2^	90.2 ± 5.9	81.4 ± 6.9	169.7 ± 7.8	266.7 ± 7.8	197.1 ± 5.9	208.9 ± 5.9	179.5 ± 5.9	188.3 ± 5.9
Friability (%) ^3^	Capping	Capping	0.07	0.02	0.03	0.02	0.06	0.05
Disintegration (min) ^4^	2.2 ± 0.2	1.7 ± 0.2	16.8 ± 0.7	25.3 ± 1.1	17.3 ± 0.6	20.2 ± 0.8	11.2 ± 2.1	7.8 ± 1.2

Notes: The MF wet granules were dried at 60 °C until the LOD was ≤2.0% (*w*/*w*) before final blending. Values in parentheses indicate wt.%. ^1^ The drug content represents the mean value of 10 tablets. ^2^ Data represent the means ± SD (*n* = 10). ^3^ Tablet friability was obtained from a single measurement of 10 tablets. ^4^ Data represent the means ± SD (*n* = 6).

**Table 3:** Effect of the PRE-F on the mechanical properties of the SG–MF-loaded BLTs.

Parameters	BLT-0.5/30	BLT-3.5/30	BLT-6.5/30
Hardness (N) ^1^	206.9 ± 7.8	199.1 ± 4.9	203.0 ± 4.9
Friability (%) ^2^	0.02	0.02	ND
Disintegration (min) ^3^	8.1 ± 0.6	7.9 ± 0.6	7.7 ± 0.9
Weight variation (RSD, %) ^4^	5.5	3.2	3.4

Notes: The PRE-F was varied from 0.5 to 6.5 kN (2.8–36.9 MPa), and the MAIN-F was fixed at 30 kN (170 MPa). BLTs were denoted as formulation-PRE-F(kN)/MAIN-F(kN). Friability was not determined due to lamination defects observed at PRE-F 6.5 kN. ^1^ Data represent the mean ± SD (*n* = 10). ^2^ Tablet friability was obtained from a single measurement of 10 tablets. ^3^ Data represent the mean ± SD (*n* = 6). ^4^ Weight variation was determined from the individual weights of 10 tablets and expressed as the relative standard deviation.

**Table 4:** Effect of the MAIN-F on the mechanical properties of the SG–MF-loaded BLTs.

Parameters	BLT-3.5/20	BLT-3.5/30	BLT-3.5/40
Appearance	Pale pink and white, oval-shaped bilayer tablet	Pale pink and white, oval-shaped bilayer tablet	Pale pink and white, oval-shaped bilayer tablet
Hardness (N) ^1^	164.8 ± 6.9	199.1 ± 4.9	238.3 ± 5.9
Friability (%) ^2^	0.02	0.02	0.02
Disintegration (min) ^3^	6.3 ± 0.7	7.9 ± 0.6	10.3 ± 0.9

Notes: The MAIN-F varied from 20 to 40 kN (113–227 MPa), and the PRE-F was fixed at 3.5 kN (19.8 MPa). BLTs were denoted as formulation-PRE-F(kN)/MAIN-F(kN). ^1^ Data represent the means ± SD (*n* = 10). ^2^ Tablet friability was obtained from a single measurement of 10 tablets. ^3^ Data represent the means ± SD (*n* = 6).

**Table 5:** Pharmacokinetic parameters of SG and MF in the plasma of healthy male subjects (*n* = 30) following oral administration of the MRP (Janumet^®^, 50/1000 mg) or the BLT.

Parameters	SG		MF	
	MRP	BLT	MRP	BLT
C_max_ (ng/mL)	150.6 ± 28.5	151.1 ± 35.8	1959.6 ± 459.8	1797.7 ± 373.1
T_max_ (h)	2.98 ± 0.81	3.10 ± 0.96	1.40 ± 0.78	1.73 ± 0.68
AUC_0–34 h_ (ng·h/mL)	1272.9 ± 180.2	1283.1 ± 158.0	12,303.7 ± 2535.1	12,208.9 ± 2441.4
T_1/2_ (h)	7.74 ± 1.12	8.32 ± 1.38	6.17 ± 1.37	6.42 ± 1.67

Notes: Data represent the means ± SD (*n* = 30).

**Table 6:** Statistical analysis of the bioequivalence between the MRP (Janumet^®^, 50/1000 mg) and the BLT in healthy male subjects (*n* = 30).

Parameters	MRP	BLT	T/R Ratio	90% CI (log 0.8–log 1.25)
**SG**				
AUC_0–34 h_ (ng·h/mL)	1272.9 ± 180.2	1283.1 ± 158.0	1.01	0.9864–1.0349
C_max_ (ng/mL)	150.6 ± 28.5	151.1 ± 35.8	0.99	0.9458–1.0440
**MF**				
AUC_0–34 h_ (ng·h/mL)	12,303.7 ± 2535.1	12,208.9 ± 2441.4	0.99	0.9440–1.0475
C_max_ (ng/mL)	1959.6 ± 459.8	1797.7 ± 373.1	0.92	0.8636–0.9833

Notes: Data represent the means ± SD (*n* = 30).

## Data Availability

The data presented in this study are contained within the article. Further inquiries can be directed to the corresponding author.

## Supplementary Materials

The following supporting information can be downloaded at: https://www.mdpi.com/article/10.3390/pharmaceutics18091066/s1, Figure S1: Visual appearance of SG–MF-loaded BLTs prepared at different PRE-Fs: (A) PRE-F of 0.5 kN and MAIN-F of 30 kN and (B) PRE-F of 3.5 kN and MAIN-F of 30 kN; Table S1: Individual plasma concentration–time data of sitagliptin (SG) following oral administration of the test product (BLT-3.5/30/2.2) in healthy male subjects; Table S2: Individual plasma concentration–time data for SG following a single oral administration of the marketed reference product (MRP) in healthy male subjects; Table S3: Individual plasma concentration–time data for metformin (MF) following a single oral administration of the test product (BLT-3.5/30/2.2) in healthy male subjects; Table S4: Individual plasma concentration–time data for MF following a single oral administration of the MRP in healthy male subjects.
