# Supplementary material for: Novel Bioequivalent Sitagliptin and Metformin Bilayer Tablet with Improved Chemical Stability
Source: Pharmaceutics. 2026 Aug 26;18(9):1066. doi: 10.3390/pharmaceutics18091066 (PMC13610732; doi:10.3390/pharmaceutics18091066)
Supplement: Supplementary file 1 [file pharmaceutics-18-01066-s001.zip › pharmaceutics-4401966-supplementary.pdf]

Supplementary Material

Novel Bioequivalent Sitagliptin and Metformin Bilayer Tablet with Improved Chemical Stability

In Gyu Yang [1,†], Jun-Young Han [1,†], Min Young Jeong [1], Dong-Wan Seo [1], Myung Joo Kang [1,*] and Sun Ho Kim [2,*]

[1]College of Pharmacy, Dankook University, 119 Dandae-ro, Dongnam-gu, Cheonan, Chungnam 31116, South Korea
[2] College of Pharmacy, Wonkwang University, 460 Iksan-daero, Iksan, Jeonbuk 54538, South Korea
[*] Correspondence: Correspondence: kangmj@dankook.ac.kr (M.J.K.); shkim0403@wku.ac.kr (S.H.K.); Tel.: +82-41-550-1446 (M.J.K.); +82-63-850-6210 (S.H.K.)
[†] These authors contributed equally to this work.

Table S1. Individual plasma concentration–time data of sitagliptin (SG) following oral administration of the test product (BLT-3.5/30/2.2) in healthy male subjects.

Subject ID	Time (h)													
	0	0.25	0.5	0.75	1	1.5	2	3	4	6	8	12	24	34
S01	0.0	28.4	46.3	45.7	45.7	49.4	61.7	192.0	129.6	77.8	58.6	35.2	11.4	6.5
S02	0.0	20.7	31.2	64.5	81.8	84.0	64.2	86.7	145.8	96.4	68.2	38.6	9.5	4.6
S03	0.0	10.2	41.2	52.9	57.4	152.5	216.3	152.5	114.2	72.1	56.6	29.4	10.8	3.4
S04	0.0	7.1	23.2	37.4	51.1	41.8	36.8	106.1	115.4	107.4	87.9	56.9	17.6	6.0
S05	0.0	1.2	14.8	39.3	54.1	89.4	164.9	153.8	127.2	91.3	76.4	43.6	12.6	4.8
S06	0.0	17.0	43.6	69.6	95.0	166.2	232.4	148.2	121.0	82.0	59.7	33.7	11.4	4.9
S07	0.0	12.7	31.9	43.0	46.1	109.8	174.2	130.9	104.9	70.2	53.6	35.5	12.4	5.9
S08	0.0	20.0	16.9	20.0	23.1	88.8	133.1	141.4	108.6	80.8	62.2	38.1	13.1	6.9
S09	0.0	3.8	20.7	31.9	49.2	62.2	67.1	90.6	114.4	72.5	50.0	28.2	10.6	1.9
S10	0.0	0.3	15.2	36.2	53.5	121.6	188.4	182.2	132.7	95.6	77.0	48.0	16.2	5.0
S11	0.0	9.0	20.7	31.2	36.2	52.3	60.3	162.4	139.5	75.8	57.2	35.6	12.5	5.0
S12	0.0	7.1	15.0	24.4	30.6	52.3	45.5	122.8	131.5	111.7	78.9	49.8	17.5	5.0
S13	0.0	1.5	21.3	33.7	50.4	62.2	71.5	142.6	140.8	96.2	64.7	36.8	11.8	5.0
S14	0.0	9.3	22.0	26.9	28.2	41.2	82.6	163.7	130.3	90.0	69.2	39.9	13.0	6.0
S15	0.0	13.1	26.3	30.6	36.2	46.7	60.3	147.0	127.2	81.4	62.8	36.2	13.1	4.8
S16	0.0	7.7	5.8	26.9	41.8	67.1	69.0	89.4	121.3	101.2	70.9	44.9	16.2	8.1
S17	0.0	14.5	42.4	64.0	64.7	65.6	137.1	132.7	95.6	63.4	50.4	29.7	8.8	3.1
S18	0.0	29.3	50.9	72.5	83.6	152.2	186.1	165.1	148.5	99.4	71.9	35.8	8.2	4.3
S19	0.0	20.7	52.9	52.3	62.2	86.9	176.1	140.2	106.7	78.3	61.6	38.0	14.9	7.5
S20	0.0	10.8	17.7	28.1	46.6	67.6	71.3	81.2	123.8	77.4	59.1	32.4	10.8	4.6
S21	0.0	4.6	14.5	25.7	40.6	81.6	161.9	132.6	105.9	68.6	54.9	34.3	13.8	8.2
S22	0.0	5.4	12.1	25.1	60.9	98.2	131.1	139.2	101.9	74.6	56.6	35.5	13.2	6.9
S23	0.0	15.1	30.5	34.3	40.4	61.6	103.2	144.7	107.5	77.7	60.9	36.7	13.8	6.9
S24	0.0	1.9	3.4	6.6	12.2	46.4	51.9	100.4	124.6	72.4	51.9	32.7	12.8	7.9
S25	0.0	28.9	36.4	38.9	50.3	78.5	126.8	139.1	105.7	73.5	59.9	35.1	14.1	6.7
S26	0.0	4.5	7.6	10.4	15.3	22.6	41.3	80.4	139.2	100.5	66.9	36.9	13.2	7.1
S27	0.0	5.7	20.6	36.7	44.8	53.5	50.4	73.4	92.6	75.2	60.3	32.4	11.3	6.3
S28	0.0	2.6	5.4	11.6	19.7	36.4	37.7	36.4	63.7	79.9	60.7	32.7	14.1	6.6
S29	0.0	5.4	25.3	27.1	40.8	56.9	100.4	117.1	84.8	57.5	46.4	30.8	13.5	8.5
S30	0.0	5.1	21.3	41.8	70.4	83.5	81.4	217.3	166.9	92.2	68.6	36.8	12.1	6.4
Mean	0	10.8	24.6	36.3	47.8	76.0	149.6	130.4	119.1	83.1	62.8	37.0	12.8	5.8
SD	0	8.4	13.6	16.5	19.4	35.3	36.1	38.8	21.0	13.4	9.4	6.3	2.3	1.6

Notes: Plasma SG concentrations are expressed in ng/mL and are presented for each individual subject at the indicated sampling times (n = 30).

Table S2. Individual plasma concentration–time data for SG following a single oral administration of the marketed reference product (MRP) in healthy male subjects.

Subject ID	Time (h)													
	**0**	**0.25**	**0.5**	**0.75**	**1**	**1.5**	**2**	**3**	**4**	**6**	**8**	**12**	**24**	**34**
S01	0.0	11.7	14.8	18.2	21.6	38.9	130.9	178.4	137.7	93.8	72.2	39.5	11.7	5.6
S02	0.0	6.7	17.6	29.9	42.9	65.7	86.1	68.8	178.7	102.8	70.7	42.3	12.9	5.8
S03	0.0	17.6	28.8	36.8	45.5	132.7	174.2	119.1	93.1	67.1	51.1	29.4	10.2	3.4
S04	0.0	8.4	32.2	39.8	43.6	53.5	64.7	88.8	105.2	90.7	67.1	43.6	13.6	5.0
S05	0.0	2.5	24.4	65.3	91.3	163.7	181.7	158.1	144.5	90.7	68.4	37.4	10.8	5.1
S06	0.0	17.2	28.2	41.8	75.8	203.9	190.9	145.1	121.6	92.5	73.9	48.0	15.0	6.9
S07	0.0	4.0	14.5	31.9	36.8	53.5	80.1	163.7	122.2	80.8	61.6	39.3	13.7	5.9
S08	0.0	7.7	24.5	29.4	30.0	43.0	122.8	143.9	109.8	78.3	62.8	38.1	12.5	2.4
S09	0.0	10.2	16.4	31.2	42.4	75.2	83.2	127.2	100.6	63.4	46.1	27.5	10.6	2.5
S10	0.0	7.1	23.2	39.3	48.0	79.5	92.5	169.2	170.0	114.2	80.8	53.5	16.9	3.8
S11	0.0	12.1	31.2	43.0	58.5	69.0	89.4	176.1	118.5	70.9	54.8	30.0	8.0	2.7
S12	0.0	5.0	10.0	14.4	17.0	28.8	78.9	145.7	131.2	99.3	77.0	46.7	18.1	6.1
S13	0.0	7.1	33.1	55.4	75.2	108.6	189.7	160.6	126.5	86.3	61.6	34.3	7.5	3.7
S14	0.0	10.6	36.8	36.8	35.5	33.0	60.5	147.0	116.6	80.1	67.1	41.8	14.9	7.0
S15	0.0	1.0	5.9	10.8	23.8	54.8	65.9	101.2	127.8	86.3	61.6	33.1	9.3	4.3
S16	0.0	20.7	21.3	36.8	49.2	61.0	78.3	117.3	161.2	96.8	70.9	40.8	13.0	5.9
S17	0.0	8.4	25.7	38.1	53.5	76.4	79.5	127.2	98.1	79.5	60.3	34.3	10.6	5.0
S18	0.0	12.7	25.6	47.2	67.6	82.3	85.5	148.5	135.5	95.4	67.0	33.0	8.7	4.3
S19	0.0	13.9	43.6	61.6	80.8	116.6	198.3	135.8	107.4	77.7	60.3	39.9	16.8	7.5
S20	0.0	6.5	17.6	24.4	36.1	53.4	65.1	128.1	104.9	75.1	55.2	31.2	10.2	4.1
S21	0.0	13.8	25.6	31.2	37.5	56.1	91.9	135.1	102.2	76.7	57.4	35.6	15.1	7.6
S22	0.0	3.2	19.4	30.5	49.2	109.4	155.3	154.7	110.1	72.7	53.5	35.5	12.5	6.9
S23	0.0	10.7	20.6	31.2	41.7	82.1	168.3	113.7	96.9	70.3	56.6	33.6	11.3	5.7
S24	0.0	9.1	15.3	17.8	37.7	76.8	77.4	77.4	113.4	76.8	58.2	32.7	12.8	7.9
S25	0.0	5.4	20.4	44.7	70.9	86.5	90.9	102.7	117.1	97.7	75.9	41.1	12.9	7.3
S26	0.0	15.7	15.1	26.3	41.3	59.4	71.9	101.2	123.6	86.8	58.1	30.7	10.1	4.5
S27	0.0	8.8	22.5	28.1	34.9	42.9	40.5	59.7	109.4	70.3	55.4	26.2	6.9	2.6
S28	0.0	9.7	16.6	22.8	30.8	59.4	105.3	97.9	87.3	60.6	48.8	29.1	9.7	4.8
S29	0.0	12.2	35.2	51.9	65.1	115.3	138.2	114.1	95.4	69.3	56.3	30.8	9.7	3.5
S30	0.0	8.8	35.6	61.7	74.8	76.9	89.7	174.9	156.9	94.1	71.7	45.5	13.8	6.4
Mean	0.0	9.6	23.4	35.9	48.6	78.6	149.6	129.4	120.8	83.2	62.7	36.8	12.0	5.1
SD	0.0	4.6	8.6	13.8	18.9	38.8	36.1	32.3	23.1	12.9	8.8	6.6	2.8	1.6

Notes: Plasma SG concentrations are expressed in ng/mL and are presented for each individual subject at the indicated sampling times (n = 30).

Table S3. Individual plasma concentration–time data for metformin (MF) following a single oral administration of the test product (BLT-3.5/30/2.2) in healthy male subjects.

Subject ID	Time(h)													
	**0**	**0.25**	**0.5**	**0.75**	**1**	**1.5**	**2**	**3**	**4**	**6**	**8**	**12**	**24**	**34**
S01	0	397.4	818.5	946.3	878.6	833.5	916.2	1021.5	705.7	495.2	314.7	134.3	51.6	21.5
S02	0.0	983.9	1532.8	1720.7	1826.0	1585.4	1404.9	1051.6	788.4	510.2	374.9	134.3	44.0	2.4
S03	0.0	344.8	856.1	886.1	983.9	968.9	1164.3	1036.5	893.7	743.3	532.8	239.5	51.6	7.3
S04	0.0	173.1	706.7	1120.1	1691.3	1781.5	1653.7	1435.8	1180.2	879.6	646.6	225.7	52.8	22.7
S05	0.0	966.0	1566.5	1814.2	1986.8	1806.6	1656.5	1273.8	981.1	733.4	470.7	163.0	35.4	20.4
S06	0.0	780.9	1570.4	2277.1	2352.3	1927.5	1938.8	1555.3	1149.3	780.9	435.0	164.3	21.5	2.1
S07	0.0	555.3	893.7	1630.5	1683.1	1660.6	1577.9	1307.2	1059.1	765.8	555.3	194.4	51.6	44.0
S08	0.0	615.5	1525.2	1562.8	1450.1	1728.2	2119.2	2051.6	1630.5	1059.1	517.7	156.8	21.5	7.6
S09	0.0	299.7	1420.0	2111.7	2186.9	2171.9	1968.8	1690.7	1254.6	916.2	762.1	247.0	55.3	14.0
S10	0.0	480.6	1104.5	1450.2	1563.0	1623.1	1788.5	1593.1	1164.6	871.5	593.3	187.4	37.1	7.0
S11	0.0	623.0	1224.5	1773.4	1577.9	2006.4	2164.3	1750.8	1570.4	1254.6	863.6	329.8	51.6	14.0

S12	0.0	608.0	1096.7	1134.3	1217.0	1743.3	1976.4	1886.1	1547.8	1126.7	600.4	194.4	47.8	7.2
S13	0.0	382.4	893.7	1307.2	1465.1	1623.0	1660.6	1450.1	1126.7	818.5	525.2	171.9	44.0	21.5
S14	0.0	638.0	690.7	841.0	1186.9	1826.0	2029.0	1773.4	1397.4	1066.6	698.2	194.4	6.4	2.2
S15	0.0	307.7	1142.1	1277.4	1427.7	1653.2	1735.9	2014.0	1863.7	1375.1	743.7	240.0	44.6	14.5
S16	0.0	720.7	1322.2	1577.9	1795.9	1769.6	1329.8	1149.3	668.1	547.8	337.3	119.2	29.0	21.5
S17	0.0	675.6	1111.7	1232.0	1329.8	1299.7	1299.7	1044.0	818.5	585.4	367.4	126.7	14.0	14.0
S18	0.0	936.1	1266.7	1717.5	2228.4	2145.8	1905.4	1567.3	1244.2	936.1	553.0	214.8	53.3	27.0
S19	0.0	388.4	1293.8	1278.9	1316.3	1421.1	1600.6	1720.4	1301.3	927.2	568.1	201.3	29.2	15.9
S20	0.0	710.5	1176.7	1718.6	2191.7	1928.6	1928.6	1763.2	1447.4	1101.5	680.5	266.9	48.9	26.3
S21	0.0	605.3	2026.3	1996.2	2094.1	2139.1	1868.4	1372.2	1086.5	763.2	454.9	154.1	33.8	26.3
S22	0.0	1297.1	1357.1	1477.4	1650.4	1830.8	1898.5	1560.2	1289.5	966.2	650.4	206.8	33.8	26.3
S23	0.0	642.9	1071.4	1372.2	1154.1	1297.9	1845.9	1462.4	1199.2	830.8	552.6	199.2	33.8	18.8
S24	0.0	605.3	830.8	996.2	1086.5	1011.3	1041.4	838.3	710.5	462.4	387.2	146.6	48.9	26.3
S25	0.0	364.7	672.9	1033.8	1943.6	2078.9	2161.7	2131.6	1958.6	1297.6	808.3	312.4	63.9	33.8
S26	0.0	304.5	988.7	1048.9	1146.6	1274.4	1394.7	1244.4	1094.9	778.2	492.5	146.6	33.8	11.3
S27	0.0	289.5	650.4	936.1	1003.8	1011.3	1063.9	1139.1	996.2	755.6	454.9	176.7	48.9	26.3
S28	0.0	800.8	1176.7	1477.4	1672.9	1672.9	1507.5	1154.1	906.1	703.6	454.9	176.7	33.8	18.8
S29	0.0	936.1	1860.9	1793.2	1770.7	1966.2	2094.1	1627.8	1319.5	951.1	650.4	274.4	56.4	33.8
S30	0.0	718.9	1199.2	973.7	928.6	1312.1	1477.4	1394.7	1154.1	943.6	597.7	206.8	41.4	26.3
Mean	0.0	612.3	1180.3	1561.7	1661.7	1721.7	1795.0	1348.3	1200.1	877.6	563.1	199.0	40.3	18.6
SD	0.0	259.3	344.1	439.6	356.9	432.7	382.0	298.1	313.9	224.2	134.4	52.3	13.5	10.4

Notes: Plasma MF concentrations are expressed in ng/mL and are presented for each individual subject at the indicated sampling times (n = 30).

Table S4. Individual plasma concentration–time data for MF following a single oral administration of the MRP in healthy male subjects.

**Subject ID**	**Time(h)**													
	**0**	**0.25**	**0.5**	**0.75**	**1**	**1.5**	**2**	**3**	**4**	**6**	**8**	**12**	**24**	**34**
S01	0	623.1	788.4	790.6	1104.2	976.4	1036.5	1299.7	968.9	653.1	412.5	156.8	51.6	21.5
S02	0.0	1044.0	1254.6	1382.4	1389.9	1224.5	998.9	811.0	668.1	465.1	292.2	111.7	29.0	7.4
S03	0.0	908.7	976.4	1141.8	1194.4	1186.9	1089.2	946.3	795.9	615.5	374.9	164.3	36.5	6.2
S04	0.0	774.3	2082.1	2548.1	2518.1	2345.2	2232.5	1946.8	1488.4	1135.1	691.7	210.6	37.8	15.2
S05	0.0	1026.1	1544.0	1866.7	1844.2	1634.0	1423.9	1176.2	906.0	628.3	463.2	230.5	80.4	35.4
S06	0.0	1329.8	2314.7	2066.6	2262.1	2059.1	2119.2	1856.1	1389.9	1029.0	585.4	209.5	29.0	6.4
S07	0.0	1029.0	1277.1	1487.6	1773.4	1856.1	1683.1	1322.2	1089.2	735.8	487.7	164.3	36.5	5.5
S08	0.0	765.8	1600.4	1795.9	1916.2	2104.2	1931.3	1607.9	1119.2	780.9	412.5	134.3	21.5	3.4
S09	0.0	1171.9	1908.7	1848.5	2006.4	2119.2	1991.4	2029.0	1675.6	1194.4	668.1	277.1	51.6	14.0
S10	0.0	856.4	1495.3	1514.1	1781.0	2126.8	2465.0	1886.2	1435.2	931.6	615.9	187.4	37.1	7.0
S11	0.0	833.5	773.4	1036.5	1442.5	1713.2	1607.9	1389.9	1111.7	833.5	517.7	217.0	44.0	14.0
S12	0.0	983.9	1728.2	2021.5	2547.8	2487.6	2006.4	1600.4	1299.7	931.3	502.7	171.9	29.0	7.0
S13	0.0	570.4	638.0	705.7	1006.4	1427.5	1352.3	1217.0	998.9	660.6	465.1	171.9	44.0	21.5
S14	0.0	743.3	961.3	1171.9	1186.9	1480.1	1893.7	2081.6	1638.0	1186.9	758.3	217.0	6.4	10.3
S15	0.0	1284.9	1871.2	1848.6	1788.5	1893.7	2059.1	2359.8	1803.5	1187.2	676.0	202.5	44.6	14.5
S16	0.0	901.2	1089.2	1495.2	1547.8	1645.5	1442.5	1149.3	826.0	623.0	382.4	119.2	29.0	21.5
S17	0.0	968.9	1367.3	1457.6	1495.2	1442.5	1397.4	1089.2	916.2	623.0	367.4	104.2	14.0	14.0
S18	0.0	1649.9	2311.1	2611.6	2754.4	2363.7	2040.6	1687.5	1221.6	891.1	530.4	214.8	27.0	19.5
S19	0.0	1017.1	1466.9	1443.5	1555.7	1817.7	1937.4	1510.9	1204.7	859.8	538.6	171.4	29.2	10.4
S20	0.0	1101.5	1349.6	1379.7	1627.8	2086.5	1913.5	1582.7	1229.3	1056.4	575.2	221.8	41.4	26.3
S21	0.0	2048.9	2672.9	2282.6	2485.7	2266.9	2018.8	1545.1	1289.5	770.7	417.3	131.6	26.3	11.3
S22	0.0	868.4	1462.4	1943.6	2522.6	2116.5	1800.8	1522.6	1131.6	853.4	530.1	146.6	41.4	11.3
S23	0.0	906.5	1560.2	1921.1	1740.6	1703.4	1515.5	1650.4	1176.7	883.5	590.2	191.7	26.3	18.8
S24	0.0	988.7	2131.6	1725.6	1597.7	1710.5	1650.4	1462.4	1274.4	883.5	582.7	229.3	48.9	33.8
S25	0.0	1109.6	2454.9	2176.7	2289.5	2011.3	1958.6	1710.5	1424.8	1018.8	703.9	259.4	48.9	18.8
S26	0.0	567.7	800.8	883.5	958.6	988.7	1048.9	1078.9	868.4	590.2	387.2	94.7	11.3	8.4

S27	0.0	695.5	1146.6	1515.1	1590.2	1417.3	1387.2	1109.9	785.7	590.2	387.2	146.6	26.3	11.3
S28	0.0	1327.1	1710.5	1755.6	1725.6	1560.2	1304.5	1056.4	793.2	560.2	387.2	124.1	33.8	11.3
S29	0.0	958.6	1297.1	1364.7	1605.3	1680.5	1733.1	1492.5	1206.8	868.4	635.3	319.5	56.4	33.8
S30	0.0	1109.7	1597.7	1379.7	1552.6	1718.9	1612.8	1462.4	1274.4	1011.3	672.9	289.5	78.9	63.9
Mean	0.0	1018.7	1546.4	1647.3	1783.0	1941.0	1710.9	1494.5	1173.9	841.3	524.1	187.4	36.8	16.6
SD	0.0	305.2	518.3	457.2	474.2	468.0	367.8	368.2	286.0	207.6	123.4	56.9	16.7	12.5

Notes: Plasma MF concentrations are expressed in ng/mL and are presented for each individual subject at the indicated sampling times (n = 30).

(a) (b)

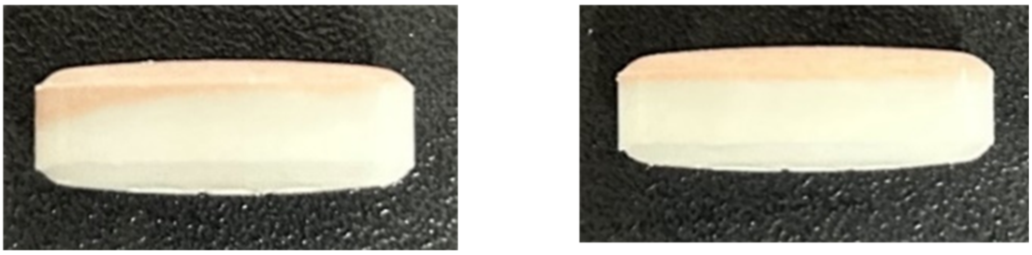

Figure S1. Visual appearance of SG–MF-loaded BLTs prepared at different PRE-Fs: (A) PRE-F of 0.5 kN and MAIN-F of 30 kN and (B) PRE-F of 3.5 kN and MAIN-F of 30 kN.
